# Iodine Clusters in the Atmosphere I: Computational Benchmark and Dimer Formation of Oxyacids and Oxides

**DOI:** 10.1021/acsomega.4c01235

**Published:** 2024-07-09

**Authors:** Morten Engsvang, Haide Wu, Jonas Elm

**Affiliations:** Department of Chemistry, Aarhus University, Langelandsgade 140, 8000 Aarhus C, Denmark

## Abstract

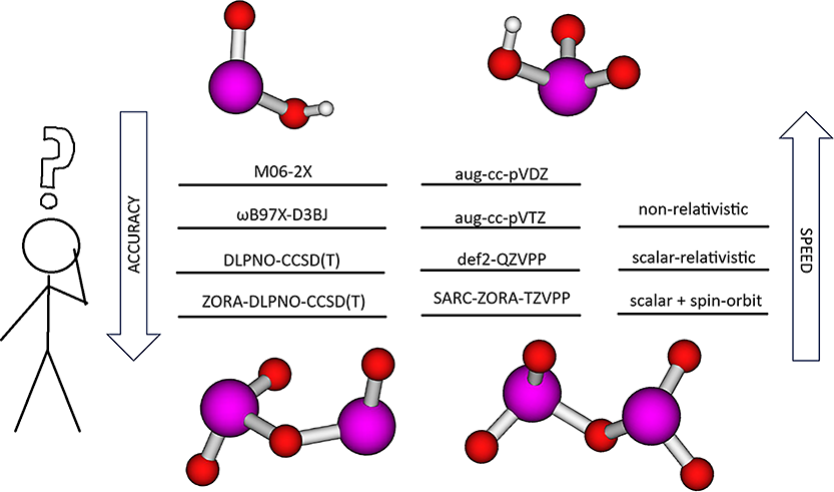

The contribution of iodine-containing compounds to atmospheric new particle formation is still not fully understood, but iodic acid and iodous acid are thought to be significant contributors. While several quantum chemical studies have been carried out on clusters containing iodine, there is no comprehensive benchmark study quantifying the accuracy of the applied methods. Here, we present the first study in a series that investigate the role of iodine species in atmospheric cluster formation. In this work, we have studied the iodic acid, iodous acid, iodine tetroxide, and iodine pentoxide monomers and their dimers formed with common atmospheric precursors. We have tested the accuracy of commonly applied methods for calculating the geometry of the monomers, thermal corrections of monomers and dimers, the contribution of spin–orbit coupling to monomers and dimers, and finally, the accuracy of the electronic energy correction calculated at different levels of theory. We find that optimizing the structures either at the ωB97X-D3BJ/aug-cc-pVTZ-PP or the M06-2X/aug-cc-pVTZ-PP level achieves the best thermal contribution to the binding free energy. The electronic energy correction can then be calculated at the ZORA-DLPNO–CCSD(T_0_) level with the SARC-ZORA-TZVPP basis for iodine and ma-ZORA-def2-TZVPP for non-iodine atoms. We applied this methodology to calculate the binding free energies of iodine-containing dimer clusters, where we confirm the qualitative trends observed in previous studies. However, we identify that previous studies overestimate the stability of the clusters by several kcal/mol due to the neglect of relativistic effects. This means that their contributions to the currently studied nucleation pathways of new particle formation are likely overestimated.

## 1 Introduction

The formation of cloud condensation nuclei (CCN) via new particle formation (NPF) is believed to account for around 50% of all CCN in the atmosphere.^1,2^ NPF starts with the formation of small clusters of gas molecules, which then grow by collision with other clusters and condensation of gases onto the clusters.^3^ However, the mechanism of their formation is still poorly understood.^4^ It has been established that the Arctic is warming two to three times faster than other parts of the globe.^5^ This warming is a result of, and will lead to changes in, aerosol precursor emissions and formation rates above the Arctic. It is important to understand the formation of aerosols since they can affect the radiative forcing in the area by the scattering of light. If the aerosols grow to between 50 and 100 nm, where they can act as CCN, they can form low-level clouds, which, especially in the arctic winter, contribute to warming due to reflection and re-emission of terrestrial radiation.^6,7^ CCN concentrations depend greatly on the season, with spring and fall dominated by the haze conditions^8^ due to anthropogenic emissions and summer dominated by natural aerosols from NPF but with low concentrations.^9^

Measurements of the initial stages of NPF are very difficult, especially if proper speciation is desired, both due to the small size of the initial clusters and the weak bonds holding the clusters together, which makes it hard to study with techniques such as mass spectrometry.^10^ Therefore, quantum chemical studies are often necessary in order to elucidate the first steps of NPF.

Iodine-containing compounds can be found in large concentrations in the atmosphere, especially in marine coastal environments but also in the Arctic and the Antarctic,^11−15^ due to large emissions from the ocean surface.^16,17^ High concentrations of iodine-containing compounds have been shown to correlate with high particle concentrations^12^ and have been shown to lead to new particle formation in the Arctic and marine environments.^12,14,18,19^ The contribution of iodine-containing compounds to NPF has previously been studied with computational methods, but the exact contribution to NPF remains poorly understood.^4^ These studies have focused primarily on the formation of clusters with iodic acid (HIO_3_) and iodous acid (HIO_2_), either by themselves^19−22^ or in combination with other known nucleation precursors such as sulfuric acid,^23^ methanesulfonic acid,^24−27^ ammonia,^28^ and dimethylamine.^29,30^ These studies show that iodic acid paired with either ammonia or dimethylamine generally forms very stable clusters, which could contribute significantly to particle formation.

The exact contribution of iodine-containing compounds is still not fully understood which is partially due to disagreements on the growth pathway. Previous studies, both computational and experimental, have suggested iodic and iodous acid pathways^12,15,18,19,21,22^ and iodine oxide pathways;^31−34^ however, a definitive conclusion is yet to be reached. Currently, very few relevant benchmark studies exist for iodine oxides such as iodine tetroxide (I_2_O_4_) and iodine pentoxide (I_2_O_5_).^35^ In addition, there are no actual benchmark studies of relevant iodine oxyacids such as iodic and iodous acids, but some high-level calculations, CCSD(T)/CBS + spin–orbit coupling, have been carried out.^36,37^ Therefore, with the increasing number of computational studies on iodine-containing compounds, calculated with many different methods (pure DFT,^28^ RI-CC2//DFT,^24,29^ and DLPNO–CCSD(T_0_)//DFT,^19,20^ all using pseudopotentials for iodine), we find it important to benchmark a subset of the methods for the most atmospherically relevant iodine-containing compounds: iodous acid HIO_2_ (IsA), iodic acid HIO_3_ (IA), iodine tetroxide I_2_O_4_ (IT), and iodine pentoxide I_2_O_5_ (IP).

Due to the weight of the iodine atoms present in these compounds, we expect relativistic effects to be relevant. Relativistic contributions can be split into scalar relativistic effects and coupling effects, most notably spin–orbit coupling. Spin–orbit coupling has been shown to be especially relevant for iodous acid,^37^ and neglecting scalar relativistic effects have been shown to yield large errors in interaction energy for some iodine-containing complexes.^38^ The commonly used pseudopotential for iodine, SK-MCDHF-RSC,^39^ has only been benchmarked for very small molecules, and the accuracy for atmospherically relevant clusters is therefore unknown.

Here, we benchmark methods for geometries, free energy thermal contributions, and single-point electronic energy contributions. This is done using either pseudopotentials for iodine atoms, scalar-relativistic methods, or scalar-relativistic methods and spin–orbit coupling.

In addition to the benchmark, we also present results for the binding free energy of dimers of IsA, IA, IT, and IP with water and various atmospherically relevant acids and bases: sulfuric acid (SA), methanesulfonic acid (MSA), nitric acid (NA), formic acid (FA), ammonia (AM), methylamine (MA), dimethylamine (DMA), trimethylamine (TMA), and ethylenediamine (EDA). This work is the first in a series of studies that investigate the role of iodine species in atmospheric cluster formation.

## 2 Computational Details

This study concerns dimers composed of either iodic acid (IA), iodous acid (IsA), iodine tetroxide (IT), or iodine pentoxide (IP) paired with either sulfuric acid (SA), methanesulfonic acid (MSA), nitric acid (NA), formic acid (FA), water (W), ammonia (AM), methylamine (MA), dimethylamine (DMA), trimethylamine (TMA), or ethylene diamine (EDA). We also include the dimer formation between the iodine-containing compounds themselves, yielding a total of 50 types of dimer clusters studied.

Unless specified otherwise in the text, all temperature- and pressure-dependent variables, such as the free energy and thermal contribution, are calculated at a standard temperature of 298.15 K and a pressure of 1 atm.

The clusters are studied using a combination of relativistic pseudopotential and scalar-relativistic methods. The pseudopotentials are used to approximate the relativistic effects. This has been shown to yield significant differences in interaction energies for some iodine-containing compounds.^38^ In this study, we use the SK-MCDHF-RSC^39^ pseudopotential for iodine, this is combined with two different types of basis sets: aug-cc-pVXZ^39,40^ and def2-QZVPP.^41^ This pseudopotential is popular; however, it has not been benchmarked in clusters and the accuracy is therefore unknown.

### 2.1 Definitions

In this study, we refer to free energies; by that term, we refer to the Gibbs free energy.

We use the following definitions of the binding free energy and the thermal contributions to the binding free energy, where *i* denotes the components of a given cluster.
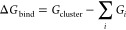
1
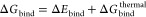
2With these definitions, we can calculate the  term at a lower level of theory and the electronic binding energy contribution Δ*E*_bind_ can be calculated at a higher level of theory.

The vibrational zero-point energy is included in the thermal contribution term, therefore electronic energies are reported without the vibrational zero-point energy correction. D_0_ values, which are the dissociation energies at zero kelvin given by the sum of electronic and zero-point energy contributions, have been computed and can be found in the Supporting Information in Section S3.4.

Due to the definition of the binding energy, the values reported here are negative if there is a favorable interaction.

We define the mean absolute error (MAE) as follows:
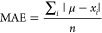
3where || denotes the absolute value, μ denotes the mean value of the data points: *x*_*i*_, *n* is the number of data points.

For the dimer energy benchmark, we use the following definition of the error:
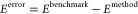
4Such that underbinding methods exhibit negative errors while overbinding methods exhibit positive errors.

### 2.2 Benchmarking Methods

All calculations, unless otherwise specified, were carried out using ORCA 5.0.3.^42^

#### 2.2.1 Geometries and Thermal Corrections for Monomers

In our geometry and thermal correction benchmark of monomers, we study the following DFT functionals: B3LYP,^43−46^ M06-2X,^47^ PW91,^48^ and ωB97X-D3BJ.^49^ This choice is based on their routine use within the field of computational atmospheric chemistry and that their accuracy is well-understood for other types of clusters.^50^

Furthermore, we study DKH–CCSD(T)^51−53^ and ZORA–CCSD(T)^54^ as possible scalar-relativistic benchmark values.

DKH here refers to calculations using the second-order approximation to the Douglas–Kroll–Hess (DKH) Hamiltonian^51−53^ and ZORA refers to calculations using the zeroth-order regular approximation (ZORA).^54^

Likewise, we also study the use of spin–orbit ZORA-B3LYP, spin–orbit ZORA-PBE0, and PBE0–4comp (full four-component relativistic DFT) as possible full-relativistic benchmark values.

The DFT methods were tested using the aug-cc-pVDZ-PP^39,40^ and aug-cc-pVTZ-PP,^39,40^ where PP denotes the use of the SK-MCDHF-RSC^39^ pseudopotential for the inner 28 electrons of iodine. For the scalar relativistic ZORA and DKH calculations, we used a combined basis of either ma-ZORA-def2-TZVPP^42^ or ma-DKH-def2-TZVPP^42^ for ZORA and DKH, respectively, for all non-iodine atoms, these are modifications of the minimally augmented Karlsruhe basis sets.^55^ SARC-ZORA-TZVPP^56^ and SARC-DKH-TZVPP^56^ all-electron basis sets were used for ZORA and DKH, respectively, to describe the iodine atoms. We denote this combination of basis sets as TZVPP.

The quasi-harmonic approximation^57^ was used in the treatment of vibrational frequencies below 100 cm^–1^.

The spin–orbit ZORA (SO-ZORA) calculations were performed with the Amsterdam density functional module (ADF)^58−60^ of the Amsterdam Modeling Suite (AMS). ZORA-adapted QZ4P Slater-type orbital basis sets were applied^61−63^ in combination with the PBE0^64−67^ and B3LYP^43−46^ functionals for both geometry optimizations and vibrational frequency calculations. A spherical Gaussian nuclear charge distribution model^68^ was applied.

The four-component relativistic calculations with the Dirac-Coulomb Hamiltonian were carried out using the DIRAC program^69^ at the DFT level with the hybrid general gradient approximation (GGA) XC-functionals B3LYP^43−46^ and PBE0.^64−67^ In these calculations, the Dyall’s v3z basis set^70^ was applied on all atoms. Symmetry was not enforced in the relativistic calculations.

#### 2.2.2 Spin–Orbit Coupling for Monomers and Dimers

For the spin–orbit coupling (SOC) stabilization energy calculations, we used time-dependent DFT (TD-DFT) with the ωB97X-D3BJ functional with the second-order approximation to the scalar relativistic DKH Hamiltonian using the aug-cc-pVQZ-DK basis set. These were carried out for different numbers of NROOTS with the TD-DFT settings of TRIPLETS TRUE, DOSOC TRUE, TDA FALSE and with relativistic settings of SOCFlags 1,4,4,0, picture change 2, FiniteNuc True. This means that it uses the second-order DKH-transformed SOC operator, with a finite nucleus model.^71^ It includes 1-electron terms, the exact Coulomb term, and exact exchange terms but without local DFT correlation due to the computation not currently being possible in ORCA.

We test the SOC stabilization energy difference upon association by calculating the SOC stabilization for the associated dimer and subtracting the SOC stabilization energy for the dissociated dimer. We simulate the dissociated dimer by moving the monomers 10 nm away from each other. Both calculations include the same number of excited states: NROOTS.

#### 2.2.3 Thermal Corrections and Single-Point Energies for Dimers

For the dimer free energy thermal correction and single-point electronic energy benchmark, we studied the same DFT functionals as used in the geometry benchmark. These were likewise carried out using the aug-cc-pVDZ-PP and aug-cc-pVTZ-PP basis sets combined with the SK-MCDHF-RSC^39^ pseudopotential for iodine. However, for the dimer thermal corrections, only the aug-cc-pVTZ-PP basis set was used.

For the single-point energies, we also studied the use of the DLPNO approximation^72^ in the form of DLPNO–CCSD(T_0_) calculations. Unless otherwise specified, all DLPNO calculations use the tight PNO criterion. We also test the DLPNO approximation in conjunction with the resolution of identity approximation^73^ denoted as RI-JK in ORCA and denoted as RIJK in this paper. The DLPNO–CCSD(T_0_) calculations used two different basis sets: aug-cc-pVTZ-PP and def2-QZVPP, using the previously mentioned SK-MCDHF-RSC^39^ and def2-ECP^39^ pseudopotentials, respectively.

The DLPNO–CCSD(T_0_) calculations were also carried out using the previously mentioned second-order DKH and ZORA Hamiltonians, using the combined basis set previously denoted as TZVPP.

The quasi-harmonic approximation^57^ was used in the treatment of vibrational frequencies below 100 cm^–1^.

The decision to study DLPNO–CCSD(T_0_) is based on its widespread use for single-point electronic energy corrections for atmospheric clusters. We try to improve it using the second-order DKH or ZORA Hamiltonians because these are two very common scalar-relatistic approximations, which are also available to us when using ORCA.

A usual concern when calculating binding energies is the emergence of basis set superposition errors (BSSE). We previously studied the basis set convergence of the binding energies of atmospherically relevant molecular clusters using the counterpoise correction (CP)^74^ and the same number of optimized parameters (SNOOP)^75^ approaches. We found that the uncorrected calculations were in better agreement with the complete basis set limit than the CP or SNOOP values when being limited to small- or medium-sized basis sets.^76−78^ This finding has been shown to be valid for both DFT,^79^ MP2,^76^ and coupled cluster methods.^76,80^ Hence, we do not correct for BSSE in this work.

### 2.3 Configurational Sampling

We follow the general workflow as set out by Kubečka et al.,^81^ where, for each cluster type, we generated an initial ensemble of structures using ABCluster^82,83^ with the CHARMM force field.^84^ This was done using a population of 3000 structures, running for 200 generations using 4 “scout bees” and with 1000 structures saved for further calculations. We employed all combinations of ionization states for the monomers in the calculations. These structures were then optimized using GFN1-xTB^85^ in the xTB program version 6.4^86^ as a pre-optimization step. A uniqueness test based on gyration radius, electronic energy, and dipole moment was used on the GFN1-xTB structures, which resulted in 20–50 unique structures for further calculations. These are chosen, such that they are as far from each other in the chemical space to avoid redundant calculations, by comparison of gyration radius, electronic energy, and dipole moment. All further calculations were carried out in ORCA 5.0.3,^42^ and for the final results, we optimized the structures at the ωB97X-D3BJ level of theory with an aug-cc-pVTZ-PP basis set for all atoms except iodine. Iodine atoms were treated using the SK-MCDHF-RSC^39^ pseudopotential for the inner 28 electrons, with the outer electrons treated with the aug-cc-pVTZ-PP basis set. In the benchmark, we also use def2-QZVPP with the corresponding pseudopotential def2-ECP for the inner 28 electrons of iodine.

For the final results, we also carried out single-point electronic energy corrections with ZORA-DLPNO–CCSD(T_0_) which indicates a DLPNO–CCSD(T_0_) calculation using the ZORA^54^ scalar relativistic Hamiltonian, where implicitly, tight PNO settings were applied for all relevant calculations both in the benchmark and the final calculation if nothing else is specified. This was carried out using the ma-ZORA-def2-TZVPP basis set for all non-iodine atoms and with the SARC-ZORA-TZVPP for iodine, which will simply be denoted as TZVPP as explained in the computational details. The quasi-harmonic approximation^57^ was used in the treatment of vibrational frequencies below 100 cm^–1^.

## 3 Results and Discussion

### 3.1 Monomer Benchmark

#### 3.1.1 Monomer Geometries

The different methods were tested to determine the similarity of the resulting geometries, which can be seen in Table 1. The geometry differences were quantified using root-mean-square deviations (RMSD), calculated using ArbAlign.^87^ We tested all four iodine compounds, oxyacids will be covered here, while the oxides are covered in Section S2.1 of the SI. For IsA and IA, we used the structures optimized at DKH–CCSD(T)/TZVPP as the reference structure.

**Table 1:** Root Mean Square Deviations (RMSD) for the Lowest Free Energy Structure of Each Monomer for a Given Method Compared to a Reference Structure^a^

method	RMSD(HIO_2_) [Å]	RMSD(HIO_3_) [Å]
B3LYP/aug-cc-pVDZ-PP	0.066	0.243
B3LYP/aug-cc-pVTZ-PP	0.029	0.104
M06-2X/aug-cc-pVDZ-PP	0.032	0.264
M06-2X/aug-cc-pVTZ-PP	0.029	0.204
PW91/aug-cc-pVDZ-PP	0.086	0.234
PW91/aug-cc-pVTZ-PP	0.050	0.149
ωB97X-D3BJ/aug-cc-pVDZ-PP	0.029	0.221
ωB97X-D3BJ/aug-cc-pVTZ-PP	0.013	0.177
ZORA-CCSD(T_0_)/TZVPP	0.008	0.060
SO-ZORA-B3LYP/QZVP	0.021	0.102
PBE0–4comp/Dyall3Z	0.010	0.068
SO-ZORA-PBE0/QZ4P	0.010	0.103

a For IsA and IA, this was chosen to be DKH–CCSD(T)/TZVPP. RMSD was calculated using ArbAlign.^87^

It can be seen that for IsA, the majority of the methods agree on the structure with RMSD values of less than 0.1 Å and the most accurate methods such as ZORA-CCSD(T), ωB97X-D3BJ/aug-cc-pVTZ-PP, or the relativistic DFT methods (SO-ZORA-B3LYP, PBE0–4comp, SO-ZORA-PBE0) achieve RMSD values close to or less than 0.01 Å. On the other hand, they agree less for IA where the majority of the DFT methods with pseudopotentials exhibit RMSD values of around 0.2 Å with the notable exception of B3LYP/aug-cc-pVTZ-PP and PW91/aug-cc-pVTZ-PP which exhibits RMSD values of 0.10 and 0.15 Å, respectively, with B3LYP/aug-cc-pVTZ-PP on par with the relativistic DFT, only beaten by ZORA-CCSD(T)/TZVPP with a RMSD of approximately 0.05 Å. Based on the monomer geometries, it seems that all the methods achieve low RMSD values and are suitable for obtaining the structures. For SA-W clusters, we usually use a minimum value of 0.38 Å for evaluating uniqueness,^88,89^ if this is also applicable for these monomers, then it can be noted that all the methods find the same lowest free energy structure for IsA and IA.

When only monomer geometries are taken into account, B3LYP/aug-cc-pVTZ-PP is observed to achieve relatively low and consistent errors compared to the reference and the relativistic methods. ωB97X-D3BJ/aug-cc-pVTZ-PP is seen to also achieve relatively low errors for the oxyacids.

#### 3.1.2 Monomer Thermal Corrections

The different methods will result not only in different geometries but will also result in different vibrational frequencies and rotational constants, therefore yielding different thermal contributions to the final free energy of a structure. The thermal contributions for IsA and IA calculated with different methods relative to the ones calculated at the DKH–CCSD(T)/TZVPP level of theory can be seen in Table 2.

**Table 2:** Unit Is kcal/mol, , MAE: Mean Absolute Error, Defined in the Definitions Section

method			MAE
B3LYP/aug-cc-pVTZ-PP	0.17	1.01	0.59
M06-2X/aug-cc-pVTZ-PP	–0.21	–0.07	0.14
PW91/aug-cc-pVTZ-PP	0.47	1.18	0.83
ωB97X-D3BJ/aug-cc-pVTZ-PP	–0.22	0.23	0.23
ZORA-CCSD(T)/TZVPP	0.03	0.90	0.47
SO-ZORA-PBE0/QZ4P	–0.18	0.86	0.52
SO-ZORA-B3LYP/QZ4P	0.12	1.38	0.75

It can be seen that the mean absolute error (MAE) for all the thermal contributions is less than 1 kcal/mol. However, a few individual thermal contribution errors are larger than 1 kcal/mol, namely, B3LYP (both PP and full relativistic) and PW91. The most accurate methods can be seen to be either M06-2X with an MAE of 0.14 kcal/mol or ωB97X-D3BJ with and MAE of 0.23 kcal/mol.

It should be noted that the relativistic methods in general seem to disagree with the result obtained by the scalar-relativistic method, DKH–CCSD(T), for IA, where they all exhibit errors between 0.86 and 1.38 kcal/mol.

Combined with the errors in the geometries (Figure S3), this indicates that either M06-2X or ωB97X-D3BJ will be the most reliable functional when calculating free energies.

### 3.2 Spin–Orbit Coupling

#### 3.2.1 Monomer Spin–Orbit Coupling

**Figure 1:**
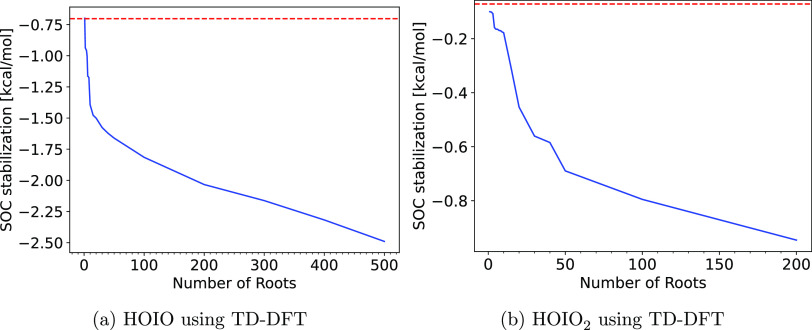
Spin–orbit coupling (SOC) stabilization of (a) HOIO and (b) HOIO_2_. TD-DFT was calculated at the DKH-ωB97X-D3BJ/aug-cc-pVQZ-DK level of theory on the structure optimized at the DKH2-CCSD(T)/def2-TZVPP level. The number of roots refers to the number of excited states included in the calculation. The results obtained by Khanniche et al. have been marked with red dashed lines.

Spin–orbit coupling (SOC) stabilization energy has previously been shown by Khanniche et al.^36,37^ to be significant for the low-energy isomer of iodous acid: HOIO. Likewise, they showed that it should be negligible for the low-energy isomer of iodic acid: HOIO_2_. The methods employed by Khanniche et al.,^36,37^ CASSCF-CASPT2 with SOC via SO-RASSI with the ANORCC-VQZP basis set on B3LYP/aug-cc-pVTZ-PP structures, are too expensive for extensive calculations; therefore, we attempt to approximate the results with TD-DFT. Figure 1, and Figure S4 in Section S2.2 of the SI, presents the convergence of the spin–orbit coupling stabilization energy, calculated at the ωB97X-D3BJ/aug-cc-pVQZ-DK level, as a function of the number of roots, which refers to the number of excited states included in the calculation. We intend to use this together with DKH-based methods, which use the second-order approximation to the relativistic Hamiltonian, where the spin–orbit operator is excluded. The TD-DFT treatment is then used to approximate the effect of the spin–orbit coupling operator within the DKH approximation.

The results by Khanniche et al.^36,37^ is shown with the dashed red lines.

It was found that the SOC results derived from TD-DFT do not appear to converge toward a fixed value with regard to the number of excited states that are included. Therefore, the predictive value of the monomer SOC is very poor. However, we are primarily interested in the change in SOC stabilization energy upon association of monomers; therefore, we investigate whether they show a better convergence in Section 3.2.2.

As the reference results are calculated on a structure optimized at a different level of theory, we tested the sensitivity of the TD-DFT calculated SOC to the structure geometry in Figures S5 and S6 in Section S2.2 of the SI. We found that it is very insensitive to geometries obtained with different methods and basis set sizes. The primary factor deciding the magnitude of the calculated SOC stabilization energy is therefore the number of excited states included in the calculation.

#### 3.2.2 Dimer Spin–Orbit Coupling

**Figure 2:**
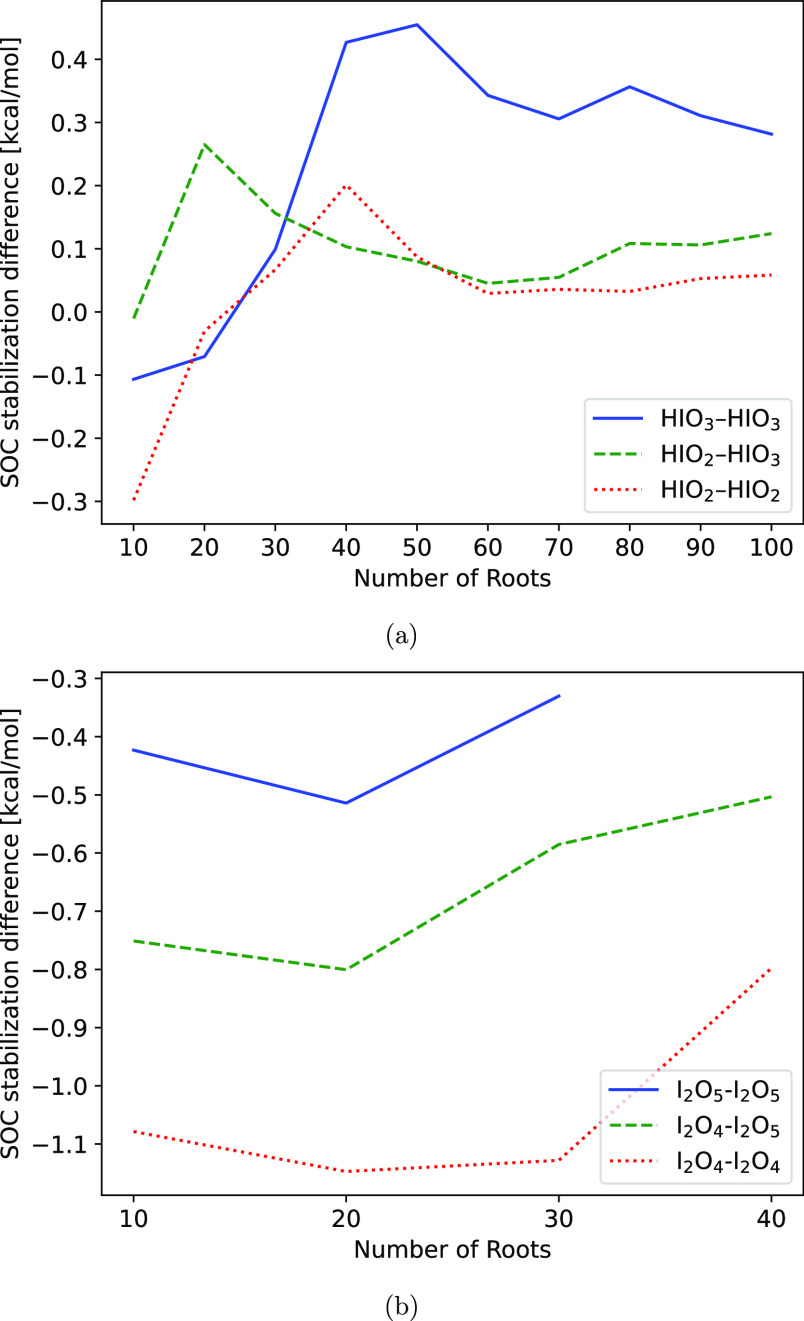
Spin–orbit coupling (SOC) stabilization energy difference upon association of the dimers. This is simulated by calculating the SOC stabilization energy for the associated dimer, and for the dissociated dimer, where they are placed 10 nm from each other. SOC is calculated at the DKH-ωB97X-D3BJ/aug-cc-pVQZ-DK level of theory on the dimer structure optimized at the ωB97X-D3BJ/aug-cc-pVTZ-PP level. (a) Convergence curves for the iodine oxyacid dimers. (b) Convergence curves for the iodine oxide dimers. This is cutoff at 30 and 40 roots due to the high memory cost of the calculations

The effect of SOC stabilization change was tested for dimers of iodine oxyacids and iodine oxides, which can be seen in Figure 2, the error is defined such that a positive error indicates that the clusters are destabilized upon association, while a negative error indicates stabilization upon association.

In Figure 2, we observe that the SOC stabilization energy difference converges toward a constant value relatively quickly. This trend is less visible for the iodine oxides, given that the memory cost of the calculations quickly became infeasible. We can conclude that using 30, ideally 40, roots in the calculation will yield decently converged results.

For the iodine oxyacid dimers shown in Figure 2a, we can observe that the stabilization energy difference converges toward negligible values for the iodous acid-containing dimers, only reaching a value of just over 0.1 kcal/mol. The iodic acid dimer reaches a value of 0.3 kcal/mol; however, compared to other expected errors, this is still negligible.

For the iodine oxides shown in Figure 2b, we see much larger changes in the SOC stabilization energy. Here, it can be observed that the dimers containing I_2_O_4_ exhibit larger changes in SOC stabilization energy than the other clusters. For the I_2_O_4_ dimer, it is on the order of −1 kcal/mol. The other clusters exhibit smaller changes, which may not be significant.

An important difference between the iodine oxyacids and the iodine oxides is that upon association we observe destabilization of the iodine oxyacid dimers, while we see a stabilization of the iodine oxides. We hypothesize that the hydrogen bonding between the iodine oxyacids reduces the degree of SOC which is possible in the dimer. We attempted to elucidate the cause further by calculating CM5 charges for the clusters, which can be seen in Table S4 in the SI. However, we do not find any clear correlation between the change in charge on the iodine atoms and the SOC change.

These SOC calculations are quite memory-intensive, especially for the iodine oxides; therefore, it may not be practically feasible to calculate this for the amount of clusters usually included in atmospheric chemistry studies. We do not include the SOC stabilization energy difference going forward; however, we will conclude that all results for the iodine oxyacids will be slight overestimations of the magnitude of the electronic binding energy, with iodic acid heavy clusters yielding higher overestimations.

Meanwhile, results for iodine oxides will yield an underestimation of the magnitude of the electronic binding energy, with I_2_O_4_ heavy clusters yielding larger underestimations.

### 3.3 Dimer Energy Benchmark

With monomer geometries, energies, and SOC stabilization corrections evaluated in the previous sections, we here wish to extend the analysis to dimers consisting of the atmospherically relevant precursors described in Section 2. While a larger ensemble of each type of dimer was created, we only carried out this benchmark for the 5 lowest free energy structures of each dimer, optimized at the ωB97X-D3BJ/aug-cc-pVTZ-PP level of theory. In this section, we use the error as defined in Section 2.1, such that under-binding methods exhibit negative errors while overbinding methods exhibit positive errors.

#### 3.3.1 Dimer Thermal Energy

**Figure 3:**
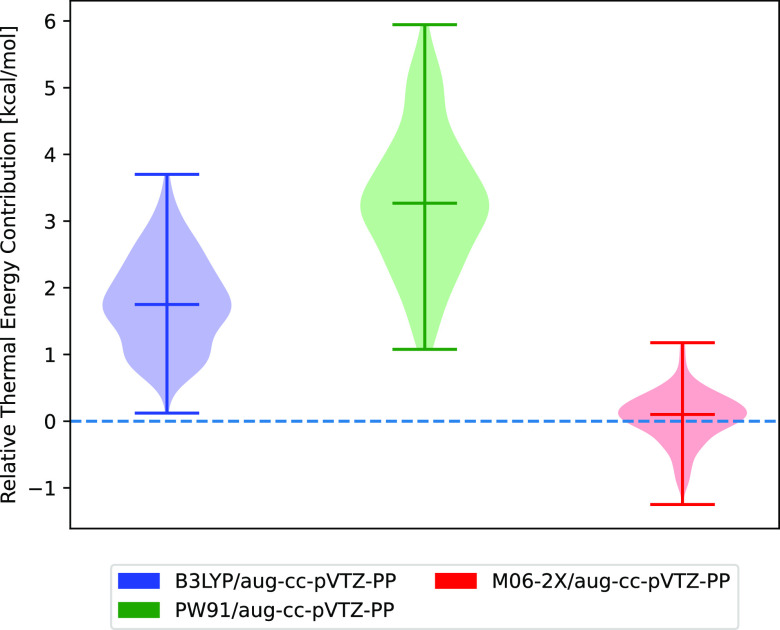
Distribution of the thermal free energy contributions relative to ωB97X-D3BJ/aug-cc-pVTZ for each structure optimized at different levels of theory. These are relative for each structure where the ωB97X-D3BJ/aug-cc-pVTZ structure was re-optimized with each level of theory.

Figure 3 shows the distribution of thermal corrections to the free energies, calculated relative to ωB97X-D3BJ/aug-cc-pVTZ, where the absolute values can be seen in Figure S7 in Section S3.1 of the SI. This reference was chosen due to its good performance compared to DKH–CCSD(T), as shown in Table 2. B3LYP and PW91 show the largest deviations with mean errors of 1.75 and 3.27 kcal/mol, respectively. Meanwhile using M06-2X leads to nearly identical thermal corrections. This means that M06-2X could be used as an alternative to ωB97X-D3BJ with no significant change in results.

#### 3.3.2 Dimer Single-Point Energy

**Figure 4:**
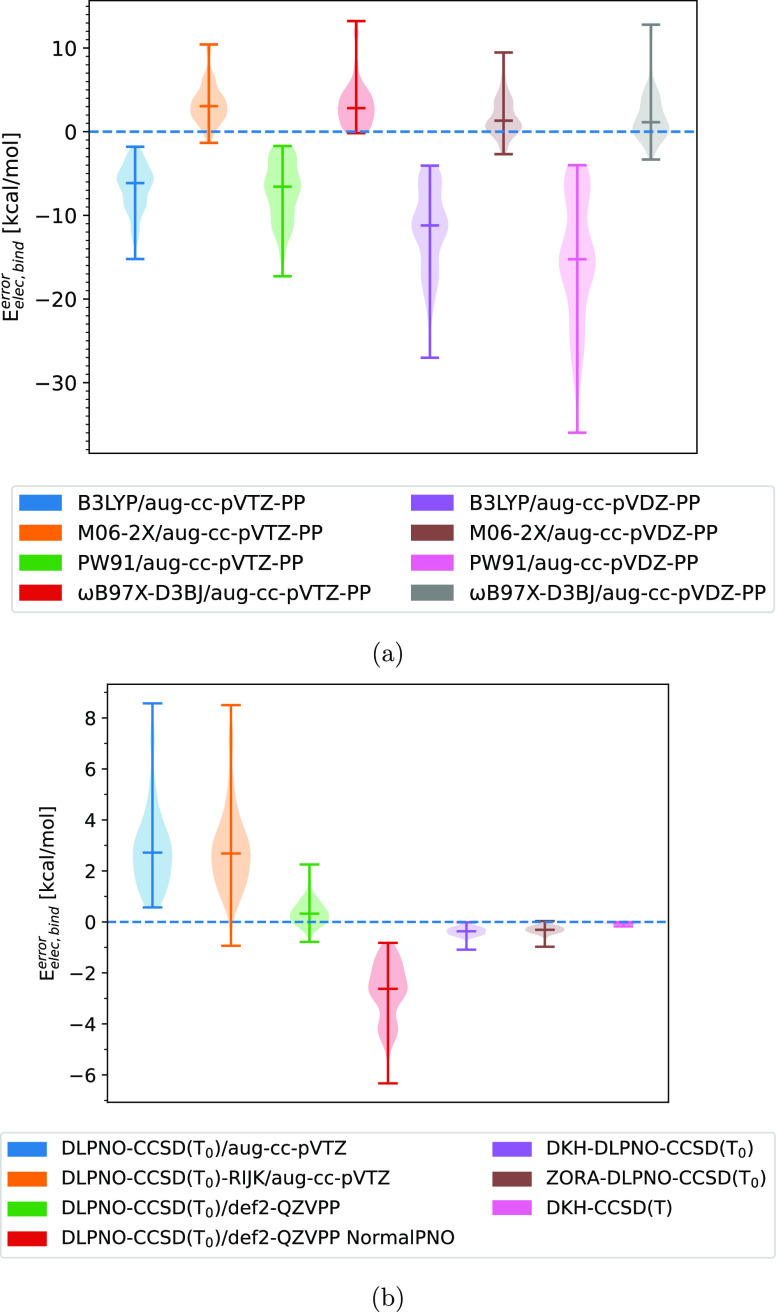
Benchmark of single-point electronic energy calculations of dimers containing either IsA, IA, IT, or IP evauluated as the electronic binding energy relative to the value calculated at the ZORA-CCSD(T)/TZVPP level of theory. All methods using DKH or ZORA use an all-electron basis set while all other methods are using a pseudopotential for I. (a) DFT methods and (b) coupled cluster-based methods; a tight PNO criterion was used unless otherwise specified.

With the thermal contributions evaluated, we will examine the electronic binding energies predicted for different single-point electronic energy methods on the ωB97X-D3BJ/aug-cc-pVTZ-PP structure. This can be seen for DFT-based methods in Figure 4a and for coupled-cluster-based methods in Figure 4b. The reference value chosen for this was ZORA-CCSD(T)/TZVPP because it was the highest level of theory for which the binding energies could be calculated for all the dimer structures. DKH–CCSD(T)/TZVPP could be carried out for the majority of the dimers; however, it proved too expensive for the largest iodine oxide dimers: IT–IP and IP–IP. In Figure 4a, it can be observed that none of the DFT methods, even those with triple ζ basis sets, predict the electronic binding energy well, with significant mean errors of several kcal/mol for all of them. Downgrading to a double ζ basis set results in shifting the energies toward underbinding, which leads to a more negative error.

This results in significant error cancellation, which means that M06-2X and ωB97X-D3BJ with a double ζ basis set give lower mean errors by several kcal/mol. On the other hand, PW91 and B3LYP, which can be seen to already underbind compared to the reference with the triple ζ basis set, are not improved and the results for these are worse.

However, none of these DFT methods are suited for electronic energy correction because the mean error is still several kcal/mol in the best case, therefore, high-level single-point electronic energy corrections are needed.

In Figure 4b, it can be seen that the relativistic pseudopotential DLPNO–CCSD(T_0_) methods, with triple ζ basis sets, are also not suitable for calculating the electronic energy correction with mean errors of up to 2.75 kcal/mol. Curiously, it can be seen that using the resolution of identity approximation (RIJK) leads to error cancellation for a small portion of the cluster structures, resulting in the distribution extending toward and past the reference value. However, the mean value is barely affected. The relativistic pseudopotential methods with quadruple ζ basis sets can be seen to yield relatively accurate values with a mean error of 0.4 kcal/mol if the tight PNO criterion is applied. However, if not, it is significantly worse, with a mean error of approximately −2.5 kcal/mol However, while the majority of the tested structures are within 1 kcal/mol, some structures reach errors up to 2.5 kcal/mol.

The scalar relativistic methods yield mean errors of less than 0.5 kcal/mol, with distribution spans of only around 1 kcal/mol ranging from approximately 0 to −1 kcal/mol error for the DLPNO-based relativistic methods. It can be seen that there is a high degree of agreement between DKH–CCSD(T) and the reference method of ZORA-CCSD(T). Due to their low mean errors, either DKH-DLPNO–CCSD(T_0_) or ZORA-DLPNO–CCSD(T_0_) are suitable choices for the electronic energy corrections.

For our calculations, we chose ZORA-DLPNO–CCSD(T_0_). We previously showed that SOC stabilization energy change could be a significant contributor to the electronic binding energy in specific cases; however, due to the computational load associated with this and the relatively small difference it makes for the dimers compared to the difference between the pseudopotentials and the scalar relativistic methods, we have refrained from doing this.

We believe that our benchmark is representative species-wise for inorganic atmospheric clusters containing iodine. Given the limited size of the cluster studied here, we can not with certainty conclude that our results are representative of much larger clusters than the ones studied here.

One should also keep in mind the size of the basis set used for the single-point electronic energy. In the SI, see Table S8, we present calculations carried out at the RI-MP2 level of theory using double-, triple-, quadruple-, and pentuple-ζ basis sets, for the minimum free energy structures identified in our calculations. These calculations are used to gauge how far our calculations are from the complete basis set (CBS) limit. We can conclude that increasing the basis set from triple-ζ to quadruple-ζ will decrease the median electronic binding energy by 0.18 kcal/mol, and increasing it further from quadruple to pentuple will decrease the median electronic binding energy by 0.20 kcal/mol further. This means that the median cluster is somewhat destabilized compared to the CBS limit. However, the largest change from triple- to quadruple-ζ is −1.04 kcal/mol and from quadruple- to pentuple-ζ, it is −1.67 kcal/mol. These large changes are observed for the oxide-containing clusters, with the clusters only containing the oxyacids exhibiting significantly smaller changes. Therefore, the limited basis set in our calculations contributes to a disproportionate overestimation of the electronic binding energy of the oxide clusters, which means that they are estimated to be relatively more unstable than the oxyacids compared to the CBS limit.

### 3.4 Iodine-Containing Dimers

Using the sampling methodology described in Section 2.3, we then obtained the lowest free energy dimer structures containing iodic and iodous acids. The calculations were performed at ZORA-DLPNO–CCSD(T_0_)/TZVPP//ωB97X-D3BJ/aug-cc-pVTZ-PP level of theory, at 298.15 K and 1 atm.

#### 3.4.1 Dimer Structures

**Figure 5:**
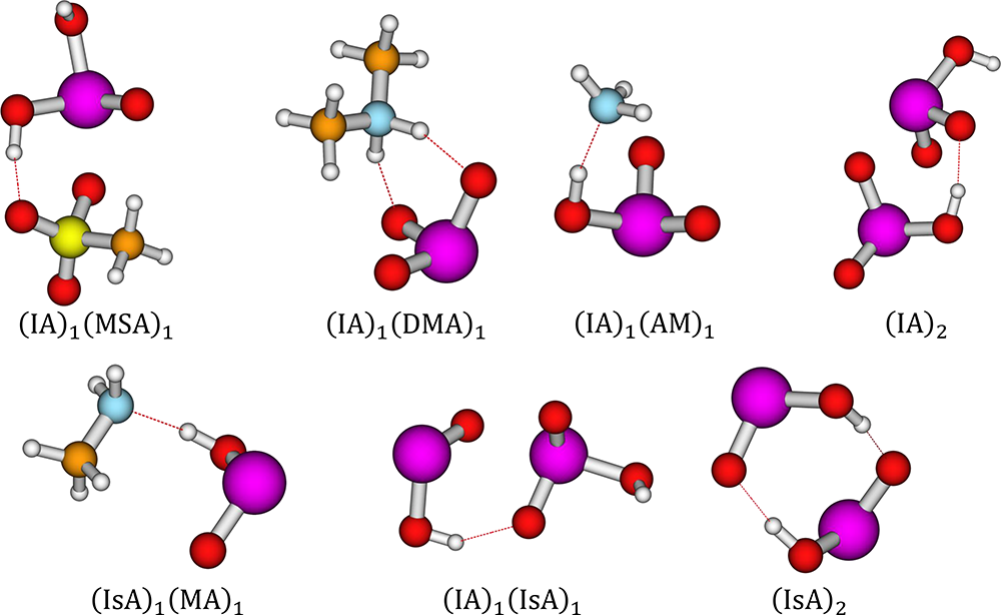
Selection of iodic and iodous acid-containing dimer clusters. Calculated at the ZORA-DLPNO–CCSD(T_0_)/TZVPP//ωB97X-D3BJ/aug-cc-pVTZ-PP level of theory. Calculated at 298.15 K and 1 atm. White = hydrogen, orange = carbon, blue = nitrogen, red = oxygen, yellow = sulfur, purple = iodine. Dashed lines indicate hydrogen bonding.

**Figure 6:**
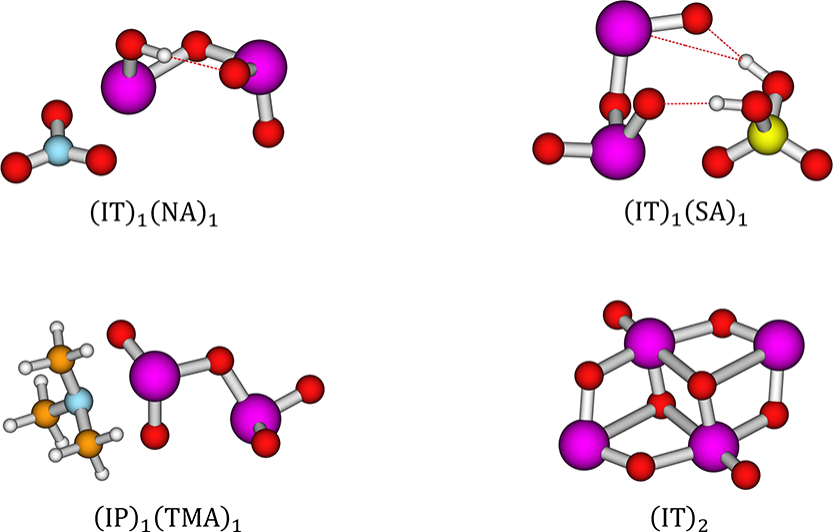
Selection of iodine oxide-containing dimer clusters. Calculated at the ZORA-DLPNO–CCSD(T_0_)/TZVPP//ωB97X-D3BJ/aug-cc-pVTZ-PP level of theory. Calculated at 298.15 K and 1 atm. White = hydrogen, orange = carbon, blue = nitrogen, red = oxygen, yellow = sulfur, purple = iodine. Dashed lines indicate hydrogen bonding.

The structures of some of the identified lowest free energy cluster structures are presented in Figures 5 and 6.

As seen in Figure 5, both iodic and iodous acids bond through hydrogen bonding; however, it can be seen that iodic acid also shows a propensity toward halogen bonds in the form of I–O coordination, as seen for (IA)_1_(MSA)_1_, (IA)_2_. (IA)_2_ structurally resembles the iodine pentoxide structure, which can be seen in Figure S1 in Section S1 of the SI. This tendency is likely due to the extra oxygen allowing for this interaction to occur side-by-side with the acid–base reaction.

Figure 6 presents the lowest free energy clusters containing iodine tetroxide (IT) and pentoxide (IP). A pattern equivalent to the one seen for iodic acid is found, where we primarily observe I–N and I–O coordination, as seen for the (IP)_1_(TMA)_1_ and (IT)_2_ clusters. However, we also observed hydrogen-bonding behavior in combination with I–N coordination, see (IT)_1_(NA)_1_ and (IT)_1_(SA)_1_. For (IT)_1_(NA)_1_, we see that iodine tetroxide is able to act as a base in the presence of nitric acid, this is also seen for (IT)_1_(FA)_1_ but not for any of the other acids. It is not observed for any of the IP clusters.

We also see the formation of a very stable iodine oxide dimer, (IT)_2_, bonded together via I–O bonds, and equivalent behavior is observed when IT is substituted for IP. Extra bonds were added to the (IT)_2_ structure based on AIM analysis performed using multiwfn 3.8^90^ In most cases, our structures match literature structures in their bonding behavior, except for the case of IA–IsA, where the DLPNO–CCSD(T_0_)/def2-QZVPP//ωB97X-D//aug-cc-pVTZ-PP level of theory^19^ finds the double hydrogen-bond configuration to be the most stable, whereas we find the hydrogen-bond + halogen bond configuration to be the most stable.

#### 3.4.2 Dimer Energies

Table 3 presents the lowest binding free energy of the obtained clusters. The clusters with the lowest binding free energy are observed to be (IT)_2_ with a binding free energy of −16.1 kcal/mol, followed by (IT)_1_(IP)_1_ at −14.6 kcal/mol. The stability of these dimers could support the hypothesis that iodine-driven nucleation can proceed through an iodine oxide polymer.^31−34^ However, due to the limited size of the clusters included in this study, no definitive conclusion should be drawn.

**Table 3:** Lowest Binding Free Energy of Iodine Containing Dimers, Given in kcal/mol, Calculated at the ZORA-DLPNO–CCSD(T_0_)/TZVPP//ωB97X-D3BJ/aug-cc-pVTZ-PP Level of Theory at 298.15 K and 1 atm^a^

	IsA	IA	IT	IP
IsA	–10.0	–9.8	–9.6	–8.1
IA		–5.8	–10.1	–6.8
IT			–16.1	–14.6
IP				–11.2
SA	–11.7	–9.6	–10.4	–8.1
MSA	–9.8	–6.1	–7.8	–4.8
NA	–5.2	–0.4	–11.6	–1.2
FA	–6.7	–3.9	–9.7	–3.4
W	–0.6	–1.0	–1.5	–0.5
AM	–1.5	–3.9	–1.2	–1.1
MA	–1.7	–3.7	–2.5	–4.9
DMA	–2.2	–7.1	–4.7	–7.5
TMA	–3.4	–7.3	–7.0	–10.3
EDA	–4.4	–6.3	–5.9	–12.4

a SOC corrections are not added to this value, and it is therefore only scalar relativistic. Rows denote the first component and columns denote the second component.

When we compare IsA and IA, we can see that IsA generally exhibits significantly lower binding free energies for bonding with other acids than is the case for IA. On the other hand, IA exhibits a larger binding affinity with bases. The higher binding affinity for IsA has previously been observed,^19,20,22^ but the lower concentration of IsA than IA by 2 orders of magnitude^12^ should be kept in mind. This binding pattern difference is likely due to the p*K*_a_ differences between IA and IsA, where IA is a significantly stronger acid than IsA, with p*K*_a_ values in the aqueous phase of 1.71^91^ for IA and 6^92^ for IsA.

The mean value of the lowest binding free energy structures of the acidic dimers is found to be −6.9 kcal/mol, while it is −5.8 kcal/mol for the basic dimers. This implies that the greater presence of oxygen, which allows I–O bonds to act as hydrogen bond donors, enhances the bonding affinity with iodine-containing clusters.

For the bases, it can be seen that in general, the binding free energy scales inversely with proton affinity, shown in parentheses, i.e., the binding free energy goes as follows: EDA (918 kJ mol^–1^)^93^ < TMA < DMA (897 kJ mol^–1^)^93^ < MA (865 kJ mol^–1^)^93^ < AM(819 kJ mol^–1^).^93^ For the acids, a trend is less clear. It can be seen that IsA and IT exhibit lower binding free energies with the acids (−11.7 to −3.2 kcal/mol) compared to IA and IP (−9.6 to −1.0 kcal/mol). This can be attributed to the binding pattern observed earlier, where IsA and IT favored hydrogen bonding while IA and IP favored halogen bonding.

Finally, the iodine species exhibit binding free energies with water ranging from −1.5 to −0.5 kcal/mol, with IA and IT exhibiting the largest affinity of −1.0 and −1.5 kcal/mol respectively. This can be compared to SA which exhibits a binding free energy with water of −1.7 kcal/mol.^88^ This means that hydration of IT is only slightly less than that of SA, while, on the other hand, IP and IsA are significantly less likely to be hydrated.

We can compare our results to the calculations by Zhang et al.,^20^ computed at 298.15 K and 1 atm at the DLPNO–CCSD(T_0_)/aug-cc-pVTZ-PP//M06-2X/6-31++G(d,p)+aug-cc-pVTZ-PP level of theory. For IA–IsA, IsA–IsA, and IA–IA, we find the binding free energy to be −9.8, −10.0, and −5.8 kcal/mol, respectively, while they find values of −16.7, −17.7, and −9.7 kcal/mol, respectively. This means that they estimate the clusters to be 4–7 kcal/mol more stable than ours, which can partly be attributed to their choice of single-point electronic energy correction of DLPNO–CCSD(T_0_)/aug-cc-pVTZ-PP, based on our benchmark results in Figure 4b.

This difference will result in lower nucleation rates for nucleation pathways only containing IA and IsA. Zhang et al. show that IA and IsA nucleation at their level of theory account for the observed results in the CLOUD chamber by He et al.^19^ However, we believe this to be due to cancellation of error. We base this on the following observations of the theoretical model by Zhang et al.^20^ and the experimental considerations by He et al.;^19^ Zhang et al. only take into account IA and IsA in their model; however, as noted by He et al.^19^ in their study, the addition of IT from the gas phase cannot be excluded given that they measure IT at 1% of the concentration of IA. Therefore, in the experiment, the concentrations of IsA and IT are approximately equal. The exact concentration of IP is, however, not commented upon but is also present. This means that the systems of clusters, that they simulate, will lead to an underestimation. There are further sources of underpredictions, such as not including water in their simulation, given that the experimental RH ranges from 34 to 73%, conformational entropy, and vibrational anharmonicity.

Thus, if one tried to make the simulation more accurate by incorporating these effects at their level of theory, then one would see very overestimated nucleation rates. Based on our benchmark, we believe that this is due to overestimation of the binding energy due to the difference between scalar relativistic calculations and pseudopotential calculations.

He et al.^19^ also studied these clusters, but at 283.15 K at the DLPNO–CCSD(T_0_)/def2-QZVPP//ωB97X-D//aug-cc-pVTZ-PP level of theory. They found the following values at 283.15 K for IA–IsA, IsA–IsA, and IA–IA: −12.9, −13.1, and −7.7 kcal/mol respectively. At the same temperature, we find −10.5, −10.7, and −6.5 kcal/mol, which can be seen in Table S9 in Section S3.5 of the SI. Therefore, they estimate the clusters to be 1.2–2.5 kcal/mol more stable than ours. This is in line with the expected overestimation of the stability when the structures are calculated at the DLPNO–CCSD(T_0_)/def2-QZVPP//ωB97X-D/aug-cc-pVTZ-PP level of theory.

This illustrates the importance of including scalar relativistic effects in order to avoid overstabilization of the iodine-containing clusters. We encourage the use of the ZORA approximation in future studies of these clusters, given its accuracy and relatively low computational cost.

Additional accuracy can be achieved by the combination of scalar relativistic effects combined with TD-DFT approximation of SOC. This improved methodology should also be applied to larger clusters in order to more accurately gauge the mechanism of iodine-driven new particle formation in the atmosphere.

## 4 Conclusions

In our benchmark, we find that thermal contributions for iodine-containing monomers are adequately described using either ωB97X-D3BJ or M06-2X with the aug-cc-pVTZ-PP basis set yielding mean absolute errors (MAE) of 0.23 and 0.14 kcal/mol, respectively.

We show that the spin–orbit coupling (SOC) stabilization energy difference of association of iodine oxyacid dimers is negligible, especially iodous acid, while it can reach a significant difference of around 1 kcal/mol for the iodine tetroxide dimer. We show that neglect of this SOC difference will lead to an overestimation of the stability of iodine oxyacid clusters and an underestimation of the stability of iodine oxide clusters.

For the dimers, we find that ωB97X-D3BJ or M06-2X with the aug-cc-pVTZ-PP basis set are also generally in agreement for the thermal contributions with maximum differences of around ±1 kcal/mol. For the single-point electronic energy, we find that none of the tested DFT methods are adequate, and the popularly used DLPNO–CCSD(T_0_) method is also inadequate if only triple ζ basis sets are used. However, if it is coupled with a scalar relativistic Hamiltonian, either the Douglass–Kroll–Hess (DKH) or the zeroth-order approximation (ZORA), we obtain results in excellent agreement with CCSD(T) with both DKH and ZORA Hamiltonians. We obtain a maximum error of less than 1.1 kcal/mol for DLPNO–CCSD(T_0_) with DKH and 1.0 kcal/mol for ZORA. We find that ZORA-DLPNO–CCSD(T_0_) is in excellent agreement with scalar-relativistic DKH–CCSD(T) corrected with the SOC stabilization energy, exhibiting a maximum error of less than 1 kcal/mol.

For the full set of generated iodine-containing dimers, we find that the acids in general bind stronger to the iodine-containing compounds compared to the bases. The lowest binding free energies of the acid dimers generally range between −4 and −12 kcal/mol, while for the bases, they range between −2 and −12 kcal/mol. Water is in general bound weakly, except for iodine tetroxide which has a binding free energy of −1.5 kcal/mol with water. The acids are all nearly equal in their ability to bind with the iodine-containing compounds, while the binding strength of the bases correlates with the proton affinity.

The dimers with the lowest binding free energy was observed to be the I_2_O_4_–I_2_O_4_ dimer with a binding free energy of −16.1 kcal/mol, followed by the I_2_O_4_–I_2_O_5_ and I_2_O_5_–I_2_O_5_ dimers.

In the following studies in this series, we will investigate larger iodine clusters using our identified methodology to fully unravel the role of iodine species in atmospheric new particle formation.

## Supporting Information Available

Structure of the I_2_O_5_ monomer, RMSD, SOC stabilization, distribution of the thermal energy contribution, median and standard deviations, binding free energies, population analysis of iodine-containing dimers, binding energies and thermochemistry of dimer clusters, and D_0_ values; structures and thermochemistries of iodine-containing clusters can be found in the ACDB^94^ which is available at https://github.com/elmjonas/ACDB/tree/master/Articles/engsvang24_iodine_benchmark (PDF)
xyz files used in the geometry benchmark and files for the RI-MP2 calculation (ZIP)

## References

[ref1] Merikanto J.; Spracklen D. V.; Mann G. W.; Pickering S. J.; Carslaw K. S. Impact of Nucleation on Global CCN. Atmos. Chem. Phys. 2009, 9, 8601–8616. 10.5194/acp-9-8601-2009.

[ref2] Gordon H.; Kirkby J.; Baltensperger U.; Bianchi F.; Breitenlechner M.; Curtius J.; Dias A.; Dommen J.; Donahue N. M.; Dunne E. M.; et al. Causes and Importance of New Particle Formation in the Present-day and Preindustrial Atmospheres. J. Geophys. Res. Atmos. 2017, 122, 8739–8760. 10.1002/2017JD026844.

[ref3] Kulmala M.; Kontkanen J.; Junninen H.; Lehtipalo K.; Manninen H. E.; Nieminen T.; Petäjä T.; Sipilä M.; Schobesberger S.; Rantala P.; et al. Direct Observations of Atmospheric Aerosol Nucleation. Science 2013, 339, 943–946. 10.1126/science.1227385. 23430652

[ref4] Engsvang M.; Wu H.; Knattrup Y.; Kubečka J.; Jensen A. B.; Elm J. Quantum Chemical Modeling of Atmospheric Molecular Clusters Involving Inorganic Acids and Methanesulfonic acid. Chem. Phys. Rev. 2023, 4, 031311 10.1063/5.0152517.

[ref5] Serreze M. C.; Barry R. G. Processes and Impacts of Arctic amplification: A Research Synthesis. Glob. Planet. Change 2011, 77, 85–96. 10.1016/j.gloplacha.2011.03.004.

[ref6] Schmale J.; Zieger P.; Ekman A. M. L. Aerosols in Current and Future Arctic Climate. Nat. Clim. Change 2021, 11, 95–105. 10.1038/s41558-020-00969-5.

[ref7] Schmale J.; Baccarini A. Progress in Unraveling Atmospheric New Particle Formation and Growth Across the Arctic. Geophys. Res. Lett. 2021, 48, e2021GL094198 10.1029/2021GL094198.

[ref8] Myhre G.; Bellouin N.; Berglen T. F.; Berntsen T. K.; Boucher O.; Grini A.; Isaksen I. S. A.; Johnsrud M.; Mishchenko M. I.; Stordal F.; et al. Comparison of the Radiative Properties and Direct Radiative Effect of Aerosols From a Global Aerosol Model and Remote Sensing Data Over Ocean. Tellus B: Chem. Phys. Meteorol. 2007, 59, 115–129. 10.1111/j.1600-0889.2006.00238.x.

[ref9] Freud E.; Krejci R.; Tunved P.; Leaitch R.; Nguyen Q. T.; Massling A.; Skov H.; Barrie L. Pan-Arctic Aerosol Number Size Distributions: Seasonality and Transport Patterns. Atmos. Chem. Phys. 2017, 17, 8101–8128. 10.5194/acp-17-8101-2017.

[ref10] Passananti M.; Zapadinsky E.; Zanca T.; Kangasluoma J.; Myllys N.; Rissanen M. P.; Kurtén T.; Ehn M.; Attoui M.; Vehkamäki H. How Well Can We Predict Cluster Fragmentation Inside a Mass Spectrometer?. Chem. Commun. 2019, 55, 5946–5949. 10.1039/C9CC02896J. 31049542

[ref11] Saiz-Lopez A.; Plane J. M. C.; Baker A. R.; Carpenter L. J.; von Glasow R.; Gómez Martín J. C.; McFiggans G.; Saunders R. W. Atmospheric Chemistry of Iodine. Chem. Rev. 2012, 112, 1773–1804. 10.1021/cr200029u. 22032347

[ref12] Sipilä M.; Sarnela N.; Jokinen T.; Henschel H.; Junninen H.; Kontkanen J.; Richters S.; Kangasluoma J.; Franchin A.; Peräkylä O.; et al. Molecular-Scale Evidence of Aerosol Particle Formation via Sequential Addition of HIO_3_. Nature 2016, 537, 532–534. 10.1038/nature19314. 27580030 PMC5136290

[ref13] Yu H.; Ren L.; Huang X.; Xie M.; He J.; Xiao H. Iodine Speciation and Size Distribution in Ambient Aerosols at a Coastal New Particle Formation Hotspot in China. Atmos. Chem. Phys. 2019, 19, 4025–4039. 10.5194/acp-19-4025-2019.

[ref14] Baccarini A.; Karlsson L.; Dommen J.; Duplessis P.; Vüllers J.; Brooks I. M.; Saiz-Lopez A.; Salter M.; Tjernström M.; Baltensperger U.; et al. Frequent New Particle Formation Over the High Arctic Pack ice by Enhanced Iodine Emissions. Nat. Commun. 2020, 11, 4924 10.1038/s41467-020-18551-0. 33004812 PMC7529815

[ref15] Finkenzeller H.; Iyer S.; He X.-C.; Simon M.; Koenig T. K.; Lee C. F.; Valiev R.; Hofbauer V.; Amorim A.; Baalbaki R.; et al. The Gas-Phase Formation Mechanism of Iodic Acid as an Atmospheric Aerosol Source. Nat. Chem. 2023, 15, 129–135. 10.1038/s41557-022-01067-z. 36376388 PMC9836935

[ref16] Garland J. A.; Curtis H. Emission of Iodine from the Sea Surface in the Presence of Ozone. J. Geophys. Res. Oceans 1981, 86, 3183–3186. 10.1029/JC086iC04p03183.

[ref17] Carpenter L. J.; MacDonald S. M.; Shaw M. D.; Kumar R.; Saunders R. W.; Parthipan R.; Wilson J.; Plane J. M. C. Atmospheric Iodine Levels Influenced by Sea Surface Emissions of Inorganic Iodine. Nat. Geosci. 2013, 6, 108–111. 10.1038/ngeo1687.

[ref18] Beck L. J.; Sarnela N.; Junninen H.; Hoppe C. J. M.; Garmash O.; Bianchi F.; Riva M.; Rose C.; Peräkylä O.; Wimmer D.; et al. Differing Mechanisms of New Particle Formation at Two Arctic Sites. Geophys. Res. Lett. 2021, 48, e2020GL091334 10.1029/2020GL091334.

[ref19] He X.-C.; Tham Y. J.; Dada L.; Wang M.; Finkenzeller H.; Stolzenburg D.; Iyer S.; Simon M.; Kürten A.; Shen J.; et al. Role of Iodine Oxoacids in Atmospheric Aerosol Nucleation. Science 2021, 371, 589–595. 10.1126/science.abe0298. 33542130

[ref20] Zhang R.; Xie H.-B.; Ma F.; Chen J.; Iyer S.; Simon M.; Heinritzi M.; Shen J.; Tham Y. J.; Kurtén T.; et al. Critical Role of Iodous Acid in Neutral Iodine Oxoacid Nucleation. Environ. Sci. Technol. 2022, 56, 14166–14177. 10.1021/acs.est.2c04328. 36126141 PMC9536010

[ref21] Zhang S.; Li S.; Ning A.; Liu L.; Zhang X. Iodous Acid – a More Efficient Nucleation Precursor than Iodic Acid. Phys. Chem. Chem. Phys. 2022, 24, 13651–13660. 10.1039/D2CP00302C. 35611676

[ref22] Liu L.; Li S.; Zu H.; Zhang X. Unexpectedly Significant Stabilizing Mechanism of Iodous Acid on Iodic Acid Nucleation under Different Atmospheric Conditions. Sci. Total Environ. 2023, 859, 159832 10.1016/j.scitotenv.2022.159832. 36404466

[ref23] He X.-C.; Simon M.; Iyer S.; Xie H.-B.; Rörup B.; Shen J.; Finkenzeller H.; Stolzenburg D.; Zhang R.; Baccarini A.; et al. Iodine Oxoacids Enhance Nucleation of Sulfuric Acid Particles in the Atmosphere. Science 2023, 382, 1308–1314. 10.1126/science.adh2526. 38096284

[ref24] Ning A.; Liu L.; Ji L.; Zhang X. Molecular-Level Nucleation Mechanism of Iodic Acid and Methanesulfonic Acid. Atmos. Chem. Phys. 2022, 22, 6103–6114. 10.5194/acp-22-6103-2022.

[ref25] Wu N.; Ning A.; Liu L.; Zu H.; Liang D.; Zhang X. Methanesulfonic Acid and Iodous Acid Nucleation: a Novel Mechanism of Marine Aerosols. Phys. Chem. Chem. Phys. 2023, 25, 16745–16752. 10.1039/D3CP01198D. 37323049

[ref26] Li J.; Wu N.; Ning A.; Zhang X. Molecular-Level Study on the Role of Methanesulfonic Acid in Iodine Oxoacids Nucleation. EGUsphere 2023, 2023, 1–17. 10.5194/egusphere-2023-2084.

[ref27] Zhang R.; Ma F.; Zhang Y.; Chen J.; Elm J.; He X.-C.; Xie H.-B. HIO 3 – HIO 2 -Driven Three-Component Nucleation: Screening Model and Cluster Formation Mechanism. Environ. Sci. Technol. 2024, 58, 649–659. 10.1021/acs.est.3c06098. 38131199

[ref28] Xia D.; Chen J.; Yu H.; Xie H.-B.; Wang Y.; Wang Z.; Xu T.; Allen D. T. Formation Mechanisms of Iodine–Ammonia Clusters in Polluted Coastal Areas Unveiled by Thermodynamics and Kinetic Simulations. Environ. Sci. Technol. 2020, 54, 9235–9242. 10.1021/acs.est.9b07476. 32589408

[ref29] Ning A.; Liu L.; Zhang S.; Yu F.; Du L.; Ge M.; Zhang X. The Critical Role of Dimethylamine in the Rapid Formation of Iodic Acid Particles in Marine Areas. npj Clim. Atmos. Sci. 2022, 5, 92 10.1038/s41612-022-00316-9.

[ref30] Zu H.; Chu B.; Lu Y.; Liu L.; Zhang X. Rapid Iodine Oxoacids Nucleation Enhanced by Dimethylamine in Broad Marine Regions. EGUsphere 2023, 2023, 1–14. 10.5194/egusphere-2023-1774.

[ref31] Gálvez O.; Gómez Martín J. C.; Gómez P. C.; Saiz-Lopez A.; Pacios L. F. A Theoretical Study on the Formation of Iodine Oxide Aggregates and Monohydrates. Phys. Chem. Chem. Phys. 2013, 15, 15572–15583. 10.1039/c3cp51219c. 23942644

[ref32] Gómez Martín J. C.; Gálvez O.; Baeza-Romero M. T.; Ingham T.; Plane J. M. C.; Blitz M. A. On The Mechanism of Iodine Oxide Particle Formation. Phys. Chem. Chem. Phys. 2013, 15, 15612–15622. 10.1039/c3cp51217g. 23942624

[ref33] Gómez Martín J. C.; Lewis T. R.; Blitz M. A.; Plane J. M. C.; Kumar M.; Francisco J. S.; Saiz-Lopez A. A Gas-to-Particle Conversion Mechanism Helps to Explain Atmospheric Particle Formation Through Clustering of Iodine Oxides. Nat. Commun. 2020, 11, 4521 10.1038/s41467-020-18252-8. 32908140 PMC7481236

[ref34] Gómez Martín J. C.; Lewis T. R.; James A. D.; Saiz-Lopez A.; Plane J. M. C. Insights into the Chemistry of Iodine New Particle Formation: The Role of Iodine Oxides and the Source of Iodic Acid. J. Am. Chem. Soc. 2022, 144, 9240–9253. 10.1021/jacs.1c12957. 35604404 PMC9164234

[ref35] Kaltsoyannis N.; Plane J. M. C. Quantum Chemical Calculations on a Selection of Iodine-Containing Species (IO, OIO, INO3, (IO)2, I2O3, I2O4 and I2O5) of Importance In The Atmosphere. Phys. Chem. Chem. Phys. 2008, 10, 1723–1733. 10.1039/b715687c. 18350176

[ref36] Khanniche S.; Louis F.; Cantrel L.; Černušák I. A Theoretical Study of the Microhydration of Iodic Acid (HOIO2). Comput. Theor. Chem. 2016, 1094, 98–107. 10.1016/j.comptc.2016.09.010.

[ref37] Khanniche S.; Louis F.; Cantrel L.; Černušák I. Thermochemistry of HIO2 Species and Reactivity of Iodous Acid with OH Radical: A Computational Study. ACS Earth Space Chem. 2017, 1, 39–49. 10.1021/acsearthspacechem.6b00010.

[ref38] Kolář M. H.; Suchá D.; Pitoňák M. Assessment of Scalar Relativistic Effects on Halogen Bonding and Sigma-hole Properties. Int. J. Quant. Chem. 2020, 120, e26392 10.1002/qua.26392.

[ref39] Peterson K. A.; Figgen D.; Goll E.; Stoll H.; Dolg M. Systematically Convergent Basis Sets with Relativistic Pseudopotentials. II. Small-Core Pseudopotentials and Correlation Consistent Basis Sets for the Post-d Group 16–18 Elements. J. Chem. Phys. 2003, 119, 11113–11123. 10.1063/1.1622924.

[ref40] Kendall R. A.; Dunning J.; Thom H.; Harrison R. J. Electron Affinities of the First-Row Atoms Revisited. Systematic Basis Sets and Wave Functions. J. Chem. Phys. 1992, 96, 6796–6806. 10.1063/1.462569.

[ref41] Weigend F.; Ahlrichs R. Balanced Basis Sets of Split Valence, Triple Zeta Valence and Quadruple Zeta Valence Quality for H to Rn: Design and Assessment of Accuracy. Phys. Chem. Chem. Phys. 2005, 7, 3297–3305. 10.1039/b508541a. 16240044

[ref42] Neese F. The ORCA Program System. Wiley Interdiscip. Rev. Comput. Mol. Sci. 2012, 2, 73–78. 10.1002/wcms.81.

[ref43] Becke A. D. Becke’s Three Parameter Hybrid Method using the LYP Correlation Functional. J. Chem. Phys. 1993, 98, 5648–5652. 10.1063/1.464913.

[ref44] Raghavachari K. Perspective on “Density Functional Thermochemistry. III. The Role of Exact Exchange. Theor. Chem. Acc. 2000, 103, 361–363. 10.1007/s002149900065.

[ref45] Miehlich B.; Savin A.; Stoll H.; Preuss H. Results Obtained with the Correlation Energy Density Functionals of Becke and Lee, Yang and Parr. Chem. Phys. Lett. 1989, 157, 200–206. 10.1016/0009-2614(89)87234-3.

[ref46] Becke A. D. Density-Functional Exchange-Energy Approximation with Correct Asymptotic Behavior. Phys. Rev. A 1988, 38, 3098 10.1103/PhysRevA.38.3098. 9900728

[ref47] Zhao Y.; Truhlar D. G. The M06 Suite of Density Functionals for Main Group Thermochemistry, Thermochemical Kinetics, Noncovalent Interactions, Excited States, and Transition Elements: Two New Functionals and Systematic Testing of Four M06-Class Functionals and 12 other Functionals. Theor. Chem. Acc. 2008, 120, 215–241. 10.1007/s00214-007-0310-x.

[ref48] Perdew J. P.; Chevary J. A.; Vosko S. H.; Jackson K. A.; Pederson M. R.; Singh D. J.; Fiolhais C. Atoms, Molecules, Solids, and Surfaces: Applications of the Generalized Gradient Approximation for Exchange and Correlation. Phys. Rev. B 1992, 46, 6671–6687. 10.1103/PhysRevB.46.6671. 10002368

[ref49] Najibi A.; Goerigk L. The Nonlocal Kernel in Van Der Waals Density Functionals as an Additive Correction: An Extensive Analysis with Special Emphasis on the B97M-V and ωB97M-V Approaches. J. Chem. Theory Comput. 2018, 14, 5725–5738. 10.1021/acs.jctc.8b00842. 30299953

[ref50] Elm J.; Kubečka J.; Besel V.; Halonen R.; Jääskeläinen M. J.; Kurtén T.; Vehkamäki H. Modeling the Formation and Growth of Atmospheric Molecular Clusters. A Review. J. Aerosol. Sci. 2020, 149, 105621 10.1016/j.jaerosci.2020.105621.

[ref51] Douglas M.; Kroll N. M. Quantum Electrodynamical Corrections to the Fine Structure of Helium. Ann. Phys. 1974, 82, 89–155. 10.1016/0003-4916(74)90333-9.

[ref52] Hess B. A. Relativistic Electronic-Structure Calculations Employing a Two-Component No-Pair Formalism with External-Field Projection Operators. Phys. Rev. A 1986, 33, 3742–3748. 10.1103/PhysRevA.33.3742. 9897114

[ref53] Jansen G.; Hess B. A. Revision of the Douglas-Kroll Transformation. Phys. Rev. A 1989, 39, 6016–6017. 10.1103/PhysRevA.39.6016. 9901188

[ref54] Lenthe E. V.; Baerends E. J.; Snijders J. G. Relativistic Regular Two-Component Hamiltonians. J. Chem. Phys. 1993, 99, 4597–4610. 10.1063/1.466059.

[ref55] Zheng J.; Xu X.; Truhlar D. G. Minimally Augmented Karlsruhe Basis Sets. Theor. Chem. Acc. 2011, 128, 295–305. 10.1007/s00214-010-0846-z.

[ref56] Rolfes J. D.; Neese F.; Pantazis D. A. All-Electron Scalar relativistic Basis Sets for the Elements Rb–Xe. J. Comput. Chem. 2020, 41, 1842–1849. 10.1002/jcc.26355. 32484577

[ref57] Grimme S. Supramolecular Binding Thermodynamics by Dispersion-Corrected Density Functional Theory. Chem.—Eur. J. 2012, 18, 9955–9964. 10.1002/chem.201200497. 22782805

[ref58] te Velde G.; Bickelhaupt F. M.; Baerends E. J.; Fonseca Guerra C.; van Gisbergen S. J. A.; Snijders J. G.; Ziegler T. Chemistry with ADF. J. Comput. Chem. 2001, 22, 931–967. 10.1002/jcc.1056.

[ref59] Baerends E. J.; Ziegler T.; Atkins A. J.; Autschbach J.; Bashford D.; Baseggio O.; Bérces A.; Bickelhaupt F. M.; Bo C.; Boerritger P. M. ADF2017, SCM, Theoretical Chemistry; Vrije Universiteit: Amsterdam, The Netherlands, https://www.scm.com, 2017.

[ref60] Guerra C. F.; Snijders J.; te Velde G. T.; Baerends E. J. Towards An Order-N DFT Method. Theor. Chem. Acc. 1998, 99, 391–403. 10.1007/s002140050353.

[ref61] Chong D. P.; Van Lenthe E.; Van Gisbergen S.; Baerends E. J. Even-Tempered Slater-Type Orbitals Revisited: From Hydrogen to Krypton. J. Comput. Chem. 2004, 25, 1030–1036. 10.1002/jcc.20030. 15067678

[ref62] Chong D. Augmenting Basis Set for Time-Dependent Density Functional Theory Calculation of Excitation Energies: Slater-Type Orbitals for Hydrogen to Krypton. Mol. Phys. 2005, 103, 749–761. 10.1080/00268970412331333618.

[ref63] Van Lenthe E.; Baerends E. J. Optimized Slater-Type Basis Sets for the Elements 1–118. J. Comput. Chem. 2003, 24, 1142–1156. 10.1002/jcc.10255. 12759913

[ref64] Perdew J. P.; Burke K.; Ernzerhof M. Generalized Gradient Approximation Made Simple. Phys. Rev. Lett. 1996, 77, 3865 10.1103/PhysRevLett.77.3865. 10062328

[ref65] Perdew J.; Burke K.; Ernzerhof M. Perdew, Burke, and Ernzerhof Reply. Phys. Rev. Lett. 1998, 80, 891 10.1103/PhysRevLett.80.891.

[ref66] Adamo C.; Barone V. Toward Reliable Density Functional Methods Without Adjustable Parameters: The PBE0Model. J. Chem. Phys. 1999, 110, 6158–6170. 10.1063/1.478522.

[ref67] Perdew J. P.; Ernzerhof M.; Burke K. Rationale for Mixing Exact Exchange with Density Functional Approximations. J. Chem. Phys. 1996, 105, 9982–9985. 10.1063/1.472933.

[ref68] Autschbach J. Magnitude of Finite-Nucleus-Size Effects in Relativistic Density Functional Computations of Indirect NMR Nuclear Spin–Spin Coupling Constants. ChemPhysChem 2009, 10, 2274–2283. 10.1002/cphc.200900271. 19670399

[ref69] Bast R.; Gomes A. S. P.; Saue T.; Visscher L. DIRAC a Relativistic Ab Initio Electronic Structure Program, Release DIRAC21. 2021, available at 10.5281/zenodo.4836496, see also http://www.diracprogram.org.

[ref70] Dyall K. G. Relativistic Double-Zeta, Triple-Zeta, and Quadruple-Zeta Basis Sets for the 6d Elements Rf–Cn. Theor. Chem. Acc. 2011, 129, 603–613. 10.1007/s00214-011-0906-z.

[ref71] Visscher L.; Dyall K. DIRAC–FOCK ATOMIC ELECTRONIC STRUCTURE CALCULATIONS USING DIFFERENT NUCLEAR CHARGE DISTRIBUTIONS. At. Data Nucl. Data Tables 1997, 67, 207–224. 10.1006/adnd.1997.0751.

[ref72] Riplinger C.; Sandhoefer B.; Hansen A.; Neese F. Natural Triple Excitations in Local Coupled Cluster Calculations with Pair Natural Orbitals. J. Chem. Phys. 2013, 139, 134101 10.1063/1.4821834. 24116546

[ref73] Weigend F.; Kattannek M.; Ahlrichs R. Approximated Electron Repulsion Integrals: Cholesky Decomposition Versus Resolution of the Identity Methods. J. Chem. Phys. 2009, 130, 164106 10.1063/1.3116103. 19405560

[ref74] Boys S. F.; Bernardi F. Calculation of Small Molecular Interactions by Differences of Separate Total Energies - Some Procedures with Reduced Errors. Mol. Phys. 1970, 19, 553 10.1080/00268977000101561.

[ref75] Kristensen K.; Ettenhuber P.; Eriksen J. J.; Jensen F.; Jo̷rgensen P. The Same Number of Optimized Parameters Scheme for Determining Intermolecular Interaction Energies. J. Chem. Phys. 2015, 142, 114116 10.1063/1.4915141. 25796240

[ref76] Elm J.; Kristensen K. Basis Set Convergence of the Binding Energies of Strongly Hydrogen-Bonded Atmospheric Clusters. Phys. Chem. Chem. Phys. 2017, 19, 1122–1133. 10.1039/C6CP06851K. 27942640

[ref77] Van Mourik T.; Wilson A. K.; Peterson K. A.; Woon D. E.; Dunning T. H. The Effect of Basis Set Superposition Error (BSSE) on the Convergence of Molecular Properties Calculated with the Correlation Consistent Basis Sets; Sabin J. R.; Zerner M. C.; Brändas E.; Wilson S.; Maruani J.; Smeyers Y.; Grout P.; McWeeny R., Eds.; Advances in Quantum Chemistry; Academic Press, 1998; Vol. 31; pp. 105–135.

[ref78] Oliphant N.; Rosenkrantz M. E.; Konowalow D. D. The Neglect of Basis Set Superposition Error in the Accurate Theoretical Determination of Heats of Formation. Chem. Phys. Lett. 1994, 223, 7–11. 10.1016/0009-2614(94)00422-6.

[ref79] Myllys N.; Elm J.; Kurtén T. Density Functional Theory Basis Set Convergence of Sulfuric Acid-Containing Molecular Clusters. Comp. Theor. Chem. 2016, 1098, 1–12. 10.1016/j.comptc.2016.10.015.

[ref80] Schmitz G.; Elm J. Assessment of the DLPNO binding energies of strongly non-covalent bonded atmospheric molecular clusters. ACS Omega 2020, 5, 7601–7612. 10.1021/acsomega.0c00436. 32280904 PMC7144154

[ref81] Kubečka J.; Besel V.; Kurtén T.; Myllys N.; Vehkamäki H. Configurational Sampling of Noncovalent (Atmospheric) Molecular Clusters: Sulfuric Acid and Guanidine. J. Phys. Chem. A 2019, 123, 6022–6033. 10.1021/acs.jpca.9b03853. 31273989

[ref82] Zhang J.; Dolg M. ABCluster: The Artificial Bee Colony Algorithm for Cluster Global Optimization. Phys. Chem. Chem. Phys. 2015, 17, 24173–24181. 10.1039/C5CP04060D. 26327507

[ref83] Zhang J.; Dolg M. Global Optimization of Clusters of Rigid Molecules Using the Artificial Bee Colony Algorithm. Phys. Chem. Chem. Phys. 2016, 18, 3003–3010. 10.1039/C5CP06313B. 26738568

[ref84] Brooks B. R.; Bruccoleri R. E.; Olafson B. D.; States D. J.; Swaminathan S.; Karplus M. CHARMM: A Program for Macromolecular Energy, Minimization, and Dynamics Calculations. J. Comput. Chem. 1983, 4, 187–217. 10.1002/jcc.540040211.

[ref85] Grimme S.; Bannwarth C.; Shushkov P. A Robust and Accurate Tight-Binding Quantum Chemical Method for Structures, Vibrational Frequencies, and Noncovalent Interactions of Large Molecular Systems Parametrized for All spd-Block Elements (Z = 1–86). J. Chem. Theory Comput. 2017, 13, 1989–2009. 10.1021/acs.jctc.7b00118. 28418654

[ref86] Bannwarth C.; Caldeweyher E.; Ehlert S.; Hansen A.; Pracht P.; Seibert J.; Spicher S.; Grimme S. Extended Tight-binding Quantum Chemistry Methods. WIREs Comput. Mol. Sci. 2021, 11, e1493 10.1002/wcms.1493.

[ref87] Temelso B.; Mabey J. M.; Kubota T.; Appiah-Padi N.; Shields G. C. ArbAlign: A Tool for Optimal Alignment of Arbitrarily Ordered Isomers Using the Kuhn–Munkres Algorithm. J. Chem. Inf. Model 2017, 57, 1045–1054. 10.1021/acs.jcim.6b00546. 28398732

[ref88] Kildgaard J. V.; Mikkelsen K. V.; Bilde M.; Elm J. Hydration of Atmospheric Molecular Clusters: A New Method for Systematic Configurational Sampling. J. Phys. Chem. A 2018, 122, 5026–5036. 10.1021/acs.jpca.8b02758. 29741906

[ref89] Kildgaard J. V.; Mikkelsen K. V.; Bilde M.; Elm J. Hydration of Atmospheric Molecular Clusters II: Organic Acid-Water Clusters. J. Phys. Chem. A 2018, 122, 8549–8556. 10.1021/acs.jpca.8b07713. 30351100

[ref90] Lu T.; Chen F. Multiwfn: A Multifunctional Wavefunction Analyzer. J. Comput. Chem. 2012, 33, 580–592. 10.1002/jcc.22885. 22162017

[ref91] Strong L. E.; Pethybridge A. D. Aqueous Iodic Acid: Conductance and Thermodynamics. J. Solution Chem. 1987, 16, 841–855. 10.1007/BF00650754.

[ref92] Schmitz G. Inorganic Reactions of Iodine(III) in Acidic Solutions and Free Energy of Iodous Acid Formation. Int. J. Chem. Kinet. 2008, 40, 647–652. 10.1002/kin.20344.

[ref93] Hunter E. P. L.; Lias S. G. Evaluated Gas Phase Basicities and Proton Affinities of Molecules: An Update. J. Phys. Chem. Ref. Data 1998, 27, 413–656. 10.1063/1.556018.

[ref94] Elm J. An Atmospheric Cluster Database Consisting of Sulfuric Acid, Bases, Organics, and Water. ACS Omega 2019, 4, 10965–10974. 10.1021/acsomega.9b00860.

