# Supplementary material for: Iodine Clusters in the Atmosphere I: Computational Benchmark and Dimer Formation of Oxyacids and Oxides
Source: ACS Omega. 2024 Jul 9;9(29):31521–32. doi: 10.1021/acsomega.4c01235 (PMC11270685; doi:10.1021/acsomega.4c01235)
Supplement: Supplementary file 1 — ao4c01235_si_001.pdf [file ao4c01235_si_001.pdf]

Iodine in the Atmosphere I: Computational Benchmark and Dimer Formation of Oxy-acids and Oxides

Morten Engsvang, Haide Wu, and Jonas Elm*

Department of Chemistry, Aarhus University, Langelandsgade 140, 8000 Aarhus C, Denmark

E-mail: jelm@chem.au.dk

Phone: +45 28938085

S1 Additional Structures

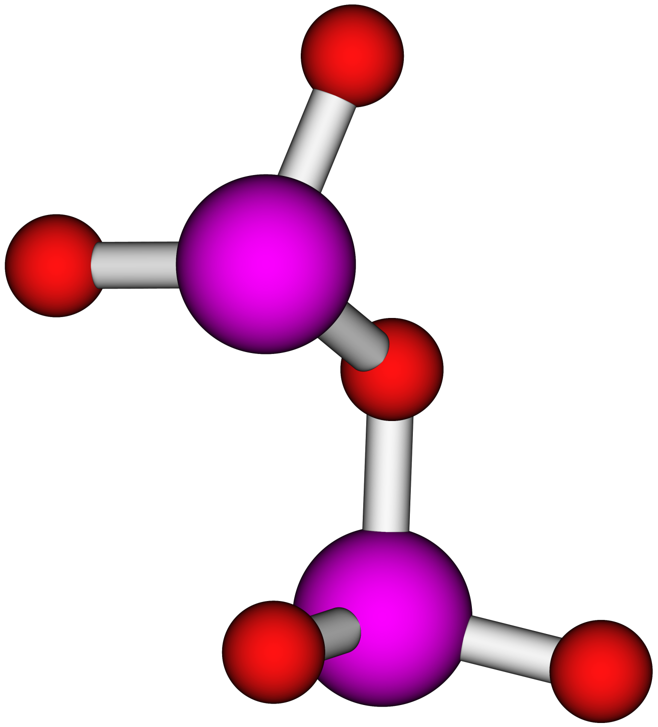

Figure S1: Structure of the I_2O_5 monomer optimized at ωB97X-D3BJ/aug-cc-pVTZ-PP level of theory

S2 Monomer Benchmark

S2.1 Monomer Geometry Benchmark

The iodine oxides used a different reference than the oxy-acids due to computational limitations. For these structures we used the ωB97X-D3BJ/aug-cc-pVTZ-PP structures as their reference. This is done because higher-level methods are not practically possible for these, therefore it can not be fully evaluated. The results of this can be seen in Figure S2

The same unambigous conclusion can not be made for for I_2O_4 and I_2O_5 where large RMSD values can be observed for the PP based DFT methods, especially when only a double ζ basis set is used, e.g. ωB97X-D3BJ/aug-cc-pVDZ-PP has an RMSD value larger than 0.7 Å for I_2O_4, this difference amounts to a roughly 90 degree rotation of one of the bonds. Increasing

the basis set to triple ζ generally lessens the geometry differences except for M06-2X/aug-cc-pVTZ-PP where the RMSD value increases from less than 0.1 Å to 0.5 Å for I_2O_5. However, the majority of the pseudo-potential DFT methods still agree on the structures of the iodine oxides.

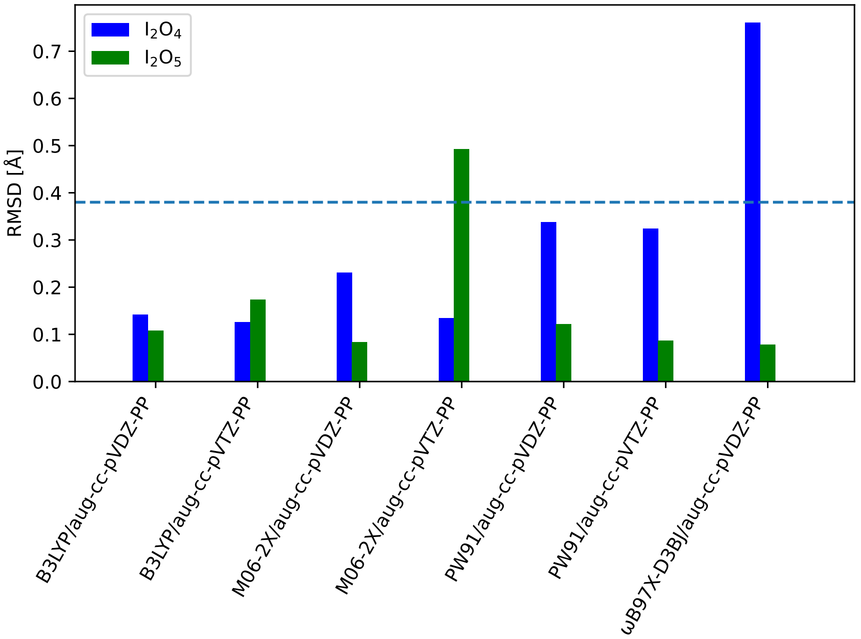

Figure S2: Root mean square deviations (RMSD) for the lowest energy structure of each monomer for a given method compared to a reference structure. For I_2O_4 and I_2O_5 it chosen to be ωB97X-D3BJ/aug-cc-pVTZ-PP. RMSD was calculated using ArbAlign. The blue dashed line at 0.38 Å is added because this value has been used in our previous studies to evaluate uniqueness of cluster structures.

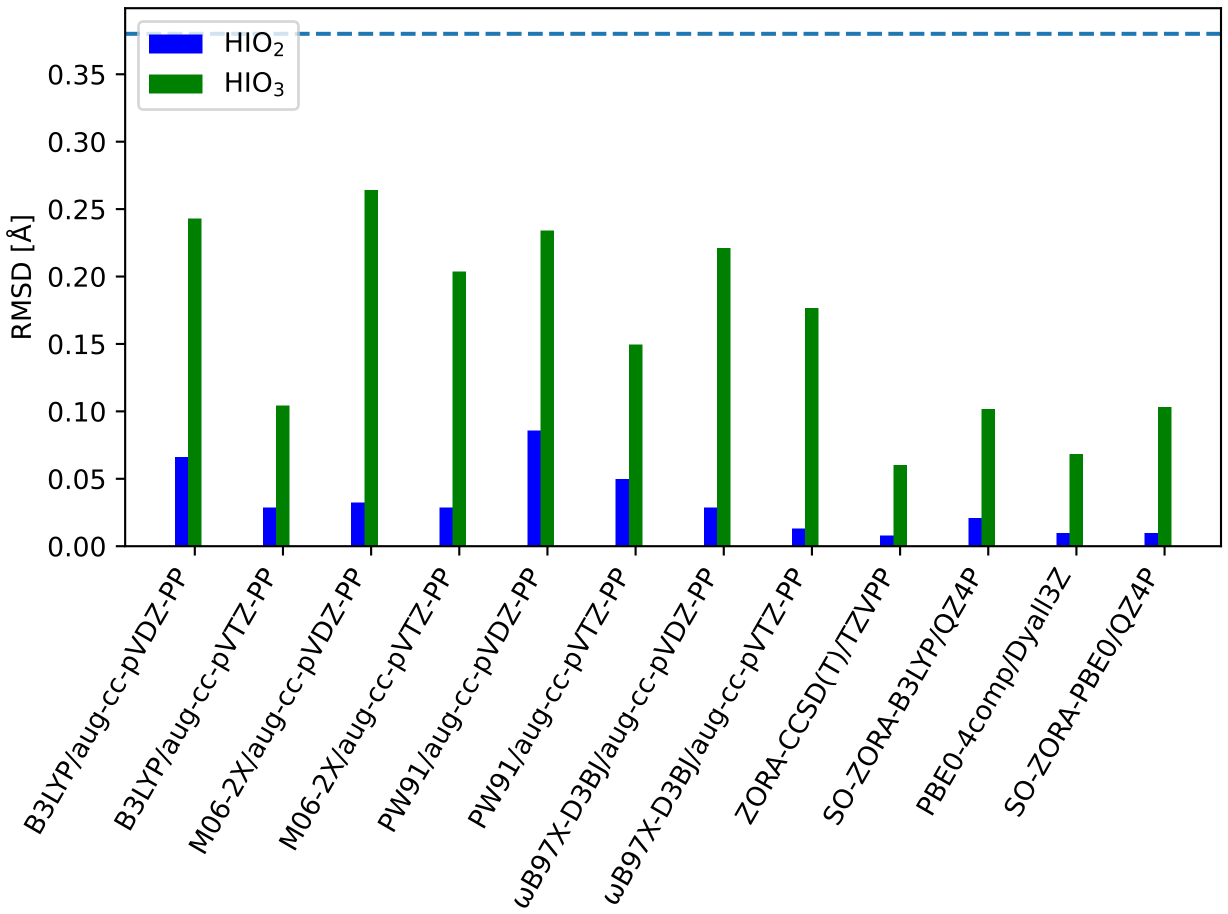

Figure S3: Root mean square deviations (RMSD) for the lowest energy structure of each monomer for a given method compared to a reference structure. For IsA and IA this was chosen to be DKH-CCSD(T)/TZVPP. RMSD was calculated using ArbAlign. The blue dashed line at 0.38 Å is added because this value has been used in our previous studies to evaluate uniqueness of cluster structures.

S2.2 Monomer Spin-orbit Coupling

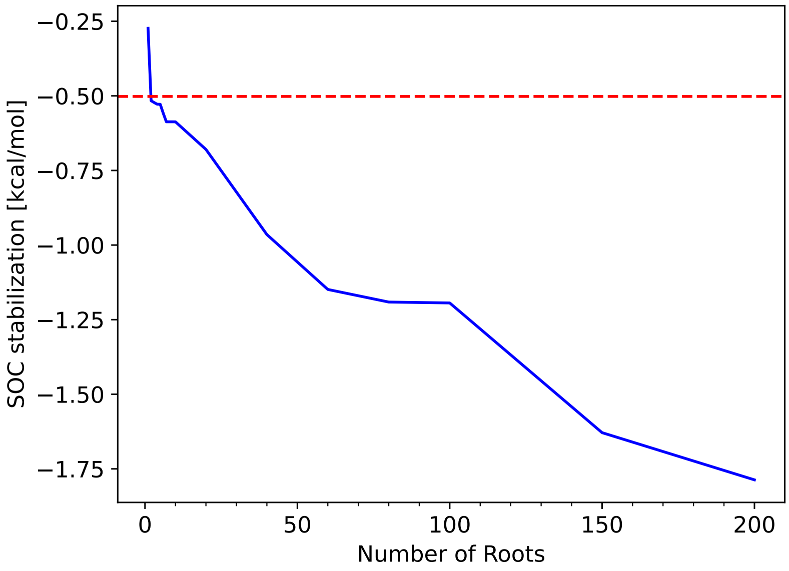

Figure S4: Spin-orbit coupling (SOC) stabilization of HI TD-DFT was calculated at the DKH-ωB97X-D3BJ/aug-cc-pVQZ-DK level of theory on the structure optimized at the DKH2-CCSD(T)/def2-TZVPP level. The number of roots refers to the number of states included in the calculation. The results obtained by Khanniche et al. have been marked with red dashed lines.

In Figure S5, the SOC stabilization energy of iodous acid, calculated at the DKH-ωB97X-D3BJ/aug-cc-pVQZ-DK level, can be seen for structures optimized at different levels of theory. It can be observed that SOC is not very sensitive to the small geometry changes due to different levels of theory for the optimization, with stabilization energies ranging from -1.35 to -1.5 kcal/mol. It should be noted that SOC for the M06-2X/aug-cc-pVTZ-PP and ωB97X-D3BJ/aug-cc-pVTZ structures best replicate the results found for the DKH-CCSD(T)/TZVPP structure. These results further support the usage of these two functionals.

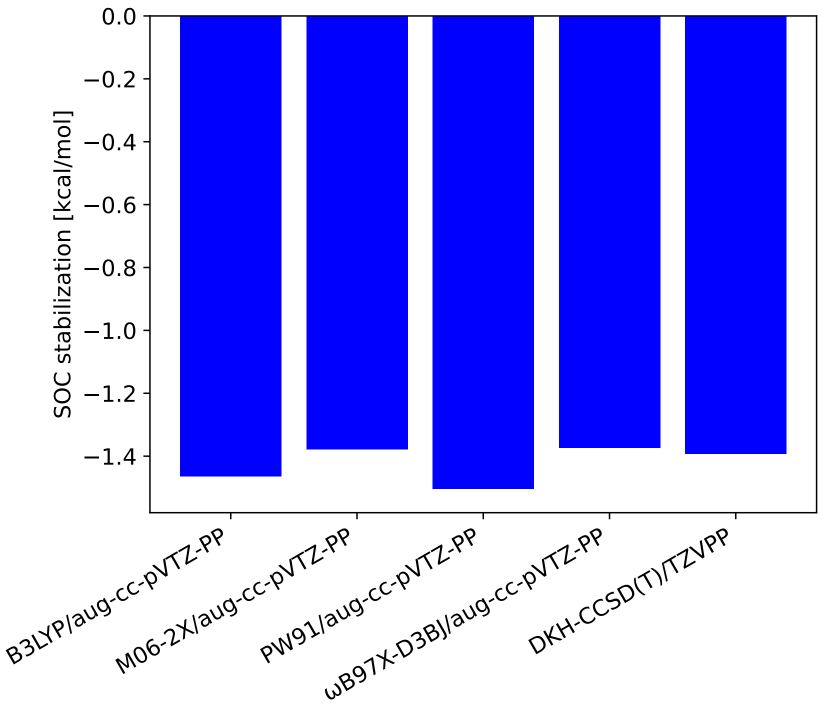

Figure S5: Spin-Orbit-Coupling (SOC) stabilization for HOIO calculated at the DKH-ωB97X-D3BJ/aug-cc-pVQZ-DK level of theory but optimized at different levels of theory. 10 states were included in the TD-DFT calculation.

We tested the sensitivity of the SOC stabilization for both HOIO and $HOIO_2$ with regards to the basis set used for the TD-DFT calculation. In Figure S6a it can be seen that the final result using 10 roots for HOIO varies between approx. −1.4 and −1.6 kcal/mol depending on the basis set used. A similar trend can be observed for all methods, where increasing the basis set size from triple ζ to quadruple ζ results in a slight decrease in the predicted SOC stabilization energy. The same can be observed for $HOIO_2$ in Figure S6b.

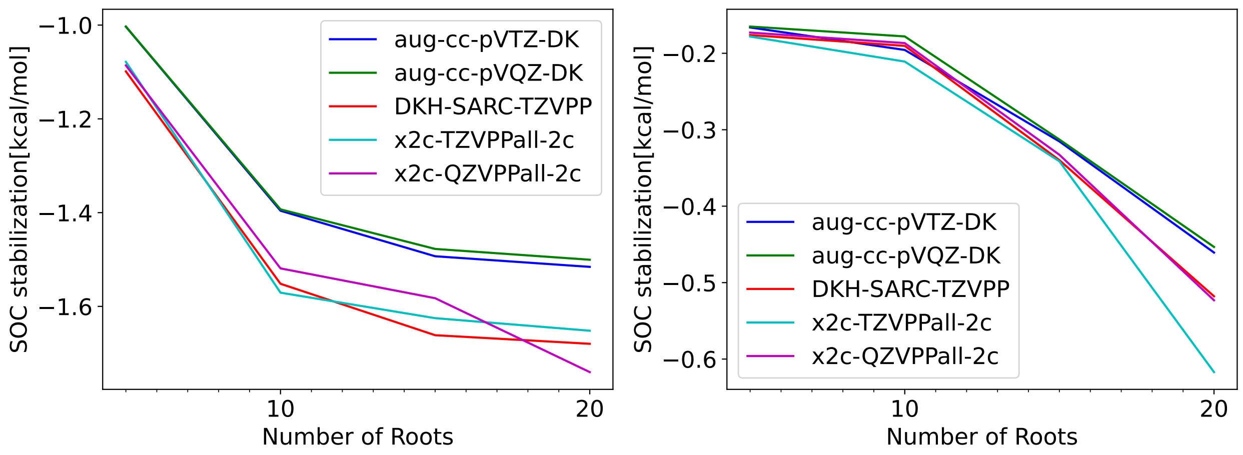

(a) HOIO

(b) $HOIO_2$

Figure S6: Spin-orbit coupling (SOC) stabilization of (a) HOIO and (b) $HOIO_2$ calculated at the DKH-ωB97X-D3BJ level of theory, with different basis sets, on the structure optimized at the DKH2-CCSD(T)/def2-TZVPP level.

S3 Dimer Energy Benchmark

S3.1 Dimer Thermal Energy

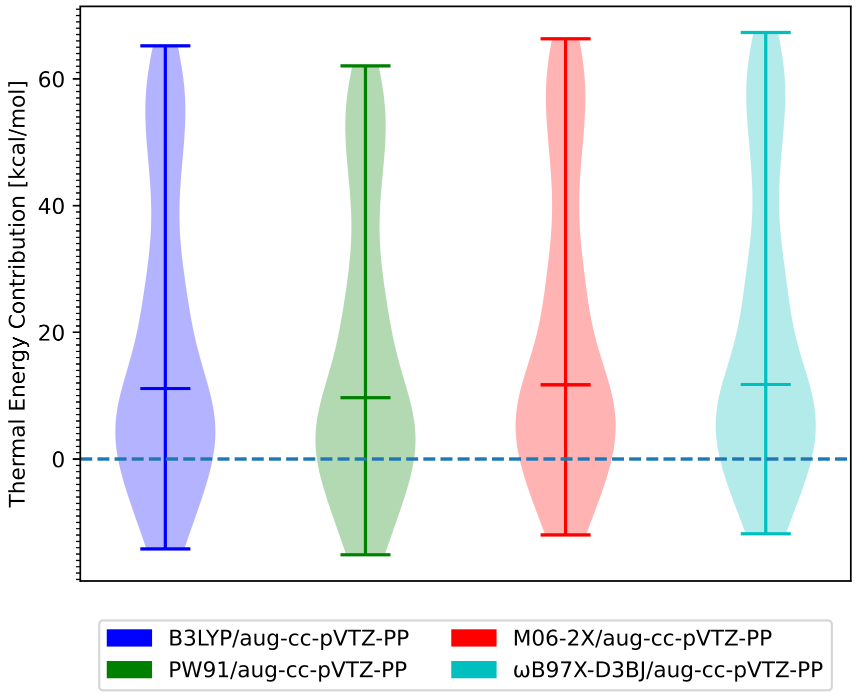

(a) Absolute distribution

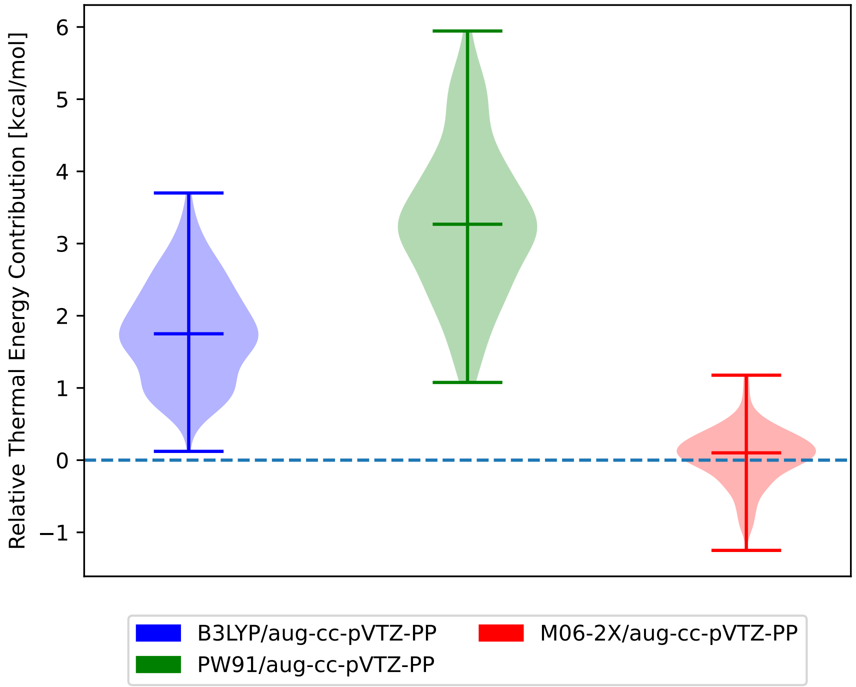

(b) Relative distribution

Figure S7: Distribution of the thermal energy contribution for each structure optimized at different levels of theory: (a) The absolute thermal contributions (b) The thermal contributions relative to ωB97X-D3BJ/aug-cc-pVTZ. These are relative for each structure where the ωB97X-D3BJ/aug-cc-pVTZ structure was re-optimized with each level of theory.

Table S1: Median and standard deviations of the distributions of thermal energy contributions at different levels of theory.

Method	Thermal Contribution [kcal/mol]
B3LYP/aug-cc-pVTZ-PP	11.1 ± 22.8
PW91/aug-cc-pVTZ-PP	9.7 ± 22.4
M06-2X/aug-cc-pVTZ-PP	11.7 ± 23.1
ωB97X-D3BJ/aug-cc-pVTZ-PP	11.8 ± 22.9

Table S2: Median and standard deviations of the distributions of relative thermal energy contributions for each structure at different levels of theory relative to ωB97X-D3BJ/aug-cc-pVTZ. These are relative for each structure where the ωB97X-D3BJ/aug-cc-pVTZ was re-optimized with each level of theory.

Method	Thermal Contribution [kcal/mol]
B3LYP/aug-cc-pVTZ-PP	1.75 ± 0.68
PW91/aug-cc-pVTZ-PP	3.27 ± 1.03
M06-2X/aug-cc-pVTZ-PP	0.10 ± 0.38

S3.2 Dimer Single-point Energy

S3.2.1 Relativistic DFT Comparison

To further evaluate the final methodology: ZORA-DLPNO-CCSD(T_0)//ωB97X-D3BJ/aug-cc-pVTZ-PP, we compare it to results derived by using 4-component relativistic DFT: ZORA-PBE0/QZ4P which can be seen in Table S3. For the relativistic DFT we only have one structure of the IsA–SA cluster, which we compare with the structures for the subset of 5 IsA–SA clusters calculated in the benchmark.

We observe excellent agreement in the binding free energy between the two methods. To our knowledge, no benchmarks have been made for thermal contributions of ZORA-PBE0/QZ4P. However, a study has been conducted benchmarking the bond-lengths against experimental data for $[Pt(CN)_4]^{2-}$,[1] where it, and other functional and basis set combinations, were shown to describe the bond-lengths within 1 pm.

Table S3: Binding free energies of selected IsA–SA dimers calculated using relativistic DFT, $G^{binding}_{Relativistic,\ DFT}$: Single-point and frequencies were calculated for an optimized structure at the PBE0/QZ4P level with SO-ZORA formalism and gaussian nuclear model in the Amsterdam Modelling Suite. For 5 optimized structures, optimized at the ωB97X-D3BJ/aug-cc-pVTZ-PP level, ZORA-DLPNO-CCSD(T_0)/TZVPP//ωB97X-D3BJ/aug-cc-pVTZ-PP was calculated: $G^{binding}_{Relativistic,\ scalar}$.

Dimer	$\Delta G^{binding}_{Relativistic,\ DFT}$ [kcal/mol]	$\Delta G^{binding}_{Relativistic,\ scalar}$ [kcal/mol]
HOIO–SA	−9.97	−9.69 ± 0.85

S3.3 Dimer SOC

Table S4: Population analysis of iodine-containing dimers. Calculated using Gaussian16 at the ωB97X-D/Def2QZVPP level. Hirshfeld population analysis was carried out to determine CM5 charges.

	Average CM5 charge on I	Total change for I
HIO_3	1.046	
HIO_2	0.615	
I_2O_5	1.062	
I_2O_4	0.853	
HIO_3–HIO_3	1.068	0.043
HIO_2–HIO_2	0.643	0.057
HIO_2–HIO_3	0.864	0.068
I_2O_4–I_2O_4	0.860	0.030
I_2O_5–I_2O_5	1.062	0.001
I_2O_4–I_2O_5	0.963	0.022

S3.4 Dimer Energies

Here we present the binding energies and thermochemistry of the identified lowest free energy clusters at the ωB97X-D3BJ/aug-cc-pVTZ-PP, and ZORA-DLPNO-CCSD(T_0)//ωB97X-D3BJ/aug-cc-pVTZ-PP level of theory. Finally we present D_0 values, which are the electronic energy with the addition of the vibrational zero-point energy.

Table S5: Binding energies and thermochemistry of dimer clusters at the ωB97X-D3BJ/aug-cc-pVTZ-PP level of theory at 298.15 K.

Cluster	ΔG[kcal/mol]	ΔH[kcal/mol]	ΔS[cal/mol/K]	ΔE[kcal/mol]
1isa1w	-1.1	-10.7	-32.4	-12.4
1ica1w	-1.2	-10.8	-32.2	-12.4
1isa1ma	-1.4	-12.8	-38.4	-14.5
1dma1isa	-1.6	-13.8	-40.9	-15.5
1am1isa	-1.9	-11.5	-32.2	-13.3
1ip1nta	-2.1	-15.3	-44.4	-16.4
1isa1tma	-2.3	-14.2	-39.8	-15.8
1ica1nta	-3.0	-16.4	-45.1	-17.1
1ip1w	-3.2	-13.2	-33.5	-14.8
1it1w	-3.8	-14.1	-34.3	-15.7
1am1it	-4.1	-14.0	-33.2	-15.7
1am1ica	-4.1	-13.9	-32.8	-15.7
1am1ip	-4.2	-14.1	-33.2	-15.8
1eda1isa	-4.2	-17.3	-44.1	-19.1
1ica1ma	-5.3	-17.2	-39.9	-19.1
1it1ma	-5.8	-18.4	-42.1	-20.3
1isa1nta	-5.9	-18.5	-42.2	-19.3
1fa1ip	-6.1	-19.3	-44.4	-20.5
1fa1ica	-6.3	-18.7	-41.6	-19.8
1ip1msa	-6.5	-21.9	-51.5	-22.8
1dma1ica	-7.5	-20.0	-42.0	-21.9

1fa1isa	-7.5	-19.9	-41.4	-21.0
1dma1it	-8.1	-21.5	-44.8	-23.5
1eda1ica	-8.2	-21.6	-45.1	-23.6
1ip1ma	-9.3	-21.9	-42.4	-23.9
1ica1tma	-9.6	-21.8	-40.9	-23.8
1isa1msa	-10.4	-24.4	-46.9	-24.6
1it1tma	-10.4	-23.6	-44.1	-25.7
1ip1sa	-10.5	-24.8	-48.3	-25.7
1ica1msa	-10.5	-25.0	-48.6	-26.0
2isa	-11.0	-24.6	-45.4	-25.7
1eda1it	-11.5	-25.8	-47.9	-27.8
1it1msa	-11.8	-26.3	-48.6	-26.2
1dma1ip	-11.8	-25.3	-45.1	-27.4
1isa1sa	-12.0	-25.3	-44.5	-25.4
1ip1isa	-12.2	-26.5	-48.0	-27.9
1it1sa	-13.4	-27.8	-48.3	-28.8
1ica1sa	-13.7	-27.6	-46.8	-28.5
2ica	-14.1	-28.1	-47.2	-29.5
1ip1tma	-14.7	-28.1	-44.8	-30.3
1fa1it	-14.8	-28.3	-45.3	-29.3
1ica1ip	-14.8	-29.3	-48.7	-30.6
1isa1it	-14.8	-29.2	-48.3	-30.6
1ica1isa	-15.3	-28.9	-45.9	-30.3
1eda1ip	-15.9	-30.9	-50.1	-33.1

1it1nta	-16.3	-30.3	-47.0	-31.2
1ica1it	-17.1	-31.6	-48.5	-32.9
2ip	-26.0	-42.1	-54.0	-43.3
1ip1it	-29.0	-45.1	-53.9	-46.3
2it	-30.4	-46.3	-53.4	-47.5

Table S6: Binding energies and thermochemistry of dimer clusters at the ZORA-DLPNO-CCSD(T_0)//ωB97X-D3BJ/aug-cc-pVTZ-PP level of theory at 298.15 K

Cluster	ΔG[kcal/mol]	ΔH[kcal/mol]	ΔS[cal/mol/K]	ΔE[kcal/mol]
1ica1nta	0.7	-12.1	-42.8	-13.2
1ip1nta	0.2	-13.1	-44.3	-14.1
1isa1w	-0.5	-10.2	-32.4	-11.9
1ica1w	-0.6	-11.1	-35.3	-12.8
1ip1w	-0.7	-10.7	-33.6	-12.3
1isa1ma	-0.8	-12.2	-38.4	-13.9
1dma1isa	-1.2	-13.4	-40.9	-15.1
1am1isa	-1.3	-10.9	-32.2	-12.7
1am1ip	-1.3	-11.3	-33.2	-13.0
1am1it	-1.7	-11.6	-33.2	-13.3
1it1w	-1.7	-12.0	-34.4	-13.6
1it1ma	-1.9	-14.4	-42.1	-16.4
1isa1tma	-2.1	-14.0	-39.7	-15.6
1fa1ip	-2.5	-15.8	-44.4	-17.0

1am1ica	-2.9	-13.3	-34.8	-15.0
1fa1ica	-3.25	-16.1	-43.2	-17.4
1ica1ma	-3.4	-16.0	-42.2	-18.4
1eda1isa	-3.7	-16.8	-44.1	-18.6
1ip1msa	-4.0	-18.3	-48.1	-19.6
1ip1ma	-4.0	-16.7	-42.5	-18.7
1dma1it	-4.0	-17.4	-44.9	-19.4
1isa1nta	-4.5	-17.1	-42.3	-17.9
1eda1ica	-5.4	-19.5	-47.2	-21.9
2ica	-5.5	-19.9	-48.1	-21.1
1ica1msa	-5.6	-20.0	-48.6	-21.0
1eda1it	-6.0	-20.3	-47.9	-22.3
1fa1isa	-6.2	-18.6	-41.4	-19.7
1it1tma	-6.3	-19.5	-44.1	-21.6
1dma1ica	-6.5	-19.6	-43.7	-22.4
1ica1tma	-6.6	-19.2	-42.4	-21.8
1dma1ip	-6.7	-20.1	-45.1	-22.2
1ica1ip	-6.7	-21.3	-48.9	-22.7
1it1msa	-7.4	-21.9	-48.8	-22.0
1ip1isa	-7.7	-22.0	-48.1	-23.4
1ip1sa	-7.9	-22.3	-48.3	-23.1
1isa1msa	-8.8	-22.8	-46.9	-23.0
2isa	-9.2	-22.7	-45.3	-23.9
1ica1sa	-9.2	-23.2	-46.8	-24.0

1ica1isa	-9.4	-23.1	-45.9	-24.4
1fa1it	-9.5	-23.0	-45.4	-24.0
1ip1tma	-9.5	-22.8	-44.8	-25.0
1ica1it	-9.7	-24.2	-48.6	-25.5
1isa1it	-10.0	-24.4	-48.3	-25.8
1it1sa	-10.1	-24.5	-48.3	-25.5
1isa1sa	-10.7	-24.0	-44.7	-24.0
1it1nta	-11.4	-25.5	-47.0	-26.4
2ip	-12.2	-28.3	-54.0	-29.4
1eda1ip	-12.7	-27.6	-50.1	-29.8
1ip1it	-15.8	-31.8	-53.9	-33.1
2it	-17.6	-33.5	-53.4	-34.7

Table S7: D_0 values (electronic energy + vibrational zero-point energy) of dimer clusters at the ZORA-DLPNO-CCSD(T_0)//ωB97X-D3BJ/aug-cc-pVTZ-PP level of theory. Defined as the reaction energy of two monomers A and B: A–B $\longrightarrow$ A + B, at zero kelvin.

Cluster	D_0[kcal/mol]
1ica1nta	12.1
1ip1nta	13.1
1isa1w	9.5
1ica1w	10.2
1ip1w	10.0
1isa1ma	12.0
1dma1isa	13.2
1am1isa	10.2

1am1ip	10.7
1am1it	11.0
1it1w	11.1
1it1ma	14.2
1isa1tma	13.9
1fa1ip	15.6
1am1ica	12.6
1fa1ica	15.7
1ica1ma	15.3
1eda1isa	16.6
1ip1msa	18.3
1ip1ma	16.4
1dma1it	17.3
1isa1nta	17.0
1eda1ica	18.8
2ica	19.4
1ica1msa	19.5
1eda1it	19.9
1fa1isa	18.2
1it1tma	19.6
1dma1ica	19.2
1ica1tma	19.0
1dma1ip	20.0
1ica1ip	21.0

1it1msa	21.1
1ip1isa	21.8
1ip1sa	21.8
1isa1msa	22.4
2isa	22.4
1ica1sa	22.6
1ica1isa	22.8
1fa1it	22.6
1ip1tma	22.7
1ica1it	24.0
1isa1it	24.2
1it1sa	24.1
1isa1sa	23.6
1it1nta	25.2
2ip	27.7
1eda1ip	26.7
1ip1it	31.2
2it	32.9

Table S8: Changes in electronic binding energy upon change in the ζ level of the basis set. Calculated on the ωB97X-D3BJ/aug-cc-pVTZ-PP structures at the RI-MP2/aug-cc-pVXZ-PP, where X is the ζ level. This is done for the identified lowest energy structure for each cluster type.

	Double-ζ $\longrightarrow$ Triple-ζ [kcal/mol]	Triple-ζ $\longrightarrow$ Quadruple-ζ [kcal/mol]
Median Value	−0.58	−0.18
Spread	0.60	0.35
Max Value	−1.96	−1.04

	Quadruple-ζ $\longrightarrow$ Pentuple-ζ [kcal/mol]
Median Value	−0.20
Spread	0.42
Max Value	−1.67

S3.5 Iodine-Containing Dimers

S3.5.1 Dimer Energies

In Figure S8, we show the full distribution and the distributions for all clusters containing either non-iodine acid (acidic), bases (basic), iodine oxy-acids (oxy-acid), or iodine oxides (oxide). Note that the distribution sizes in the violin plots are not re-scaled to the different number of points in this plot and the following plots of the results. Therefore, magnitude can be compared between different "violins" Here it can be seen that the acidic dimers in general exhibit stronger binding (median: −5.1), compared to the basic dimers (median: −1.3). According to the section on "Dimer Structures", this could be ascribed their greater ability to form hydrogen bonds and greater presence of oxygen, which can form I–O bonds.

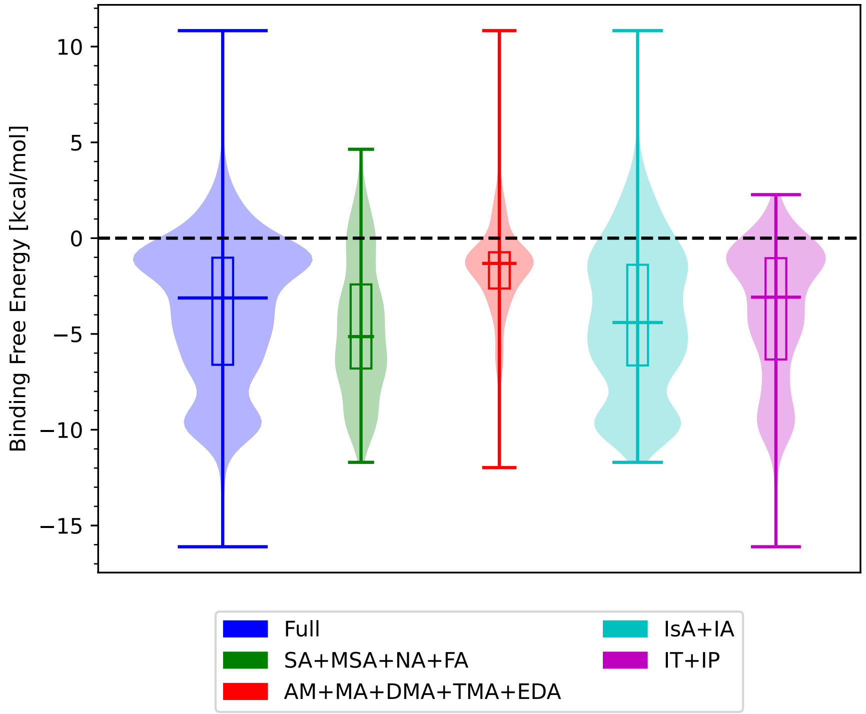

Figure S8: ZORA-DLPNO-CCSD(T_0)/TZVPP//ωB97X-D3BJ/aug-cc-pVTZ-PP binding free energies for the generated structures. The full distribution of binding free energies is plotted together with the combined distributions of dimers containing the acids: SA, MSA, NA, FA, the bases: AM, MA, DMA, TMA, EDA and iodine oxy-acids and lastly the distribution of dimers containing the iodine oxides.

More specific distributions for each type of monomers can be found in the following figures:

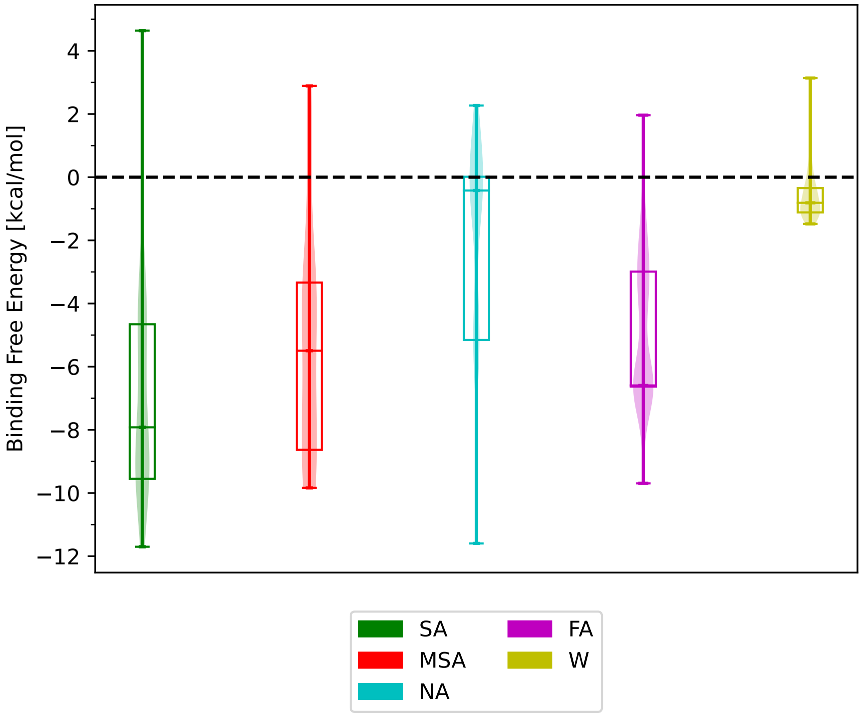

Figure S9: ZORA-DLPNO-CCSD(T_0)/TZVPP//ωB97X-D3BJ/aug-cc-pVTZ-PP binding free energies for the generated structures. The full distribution of binding free energies is plotted together with the distributions for dimers containing each acid or water.

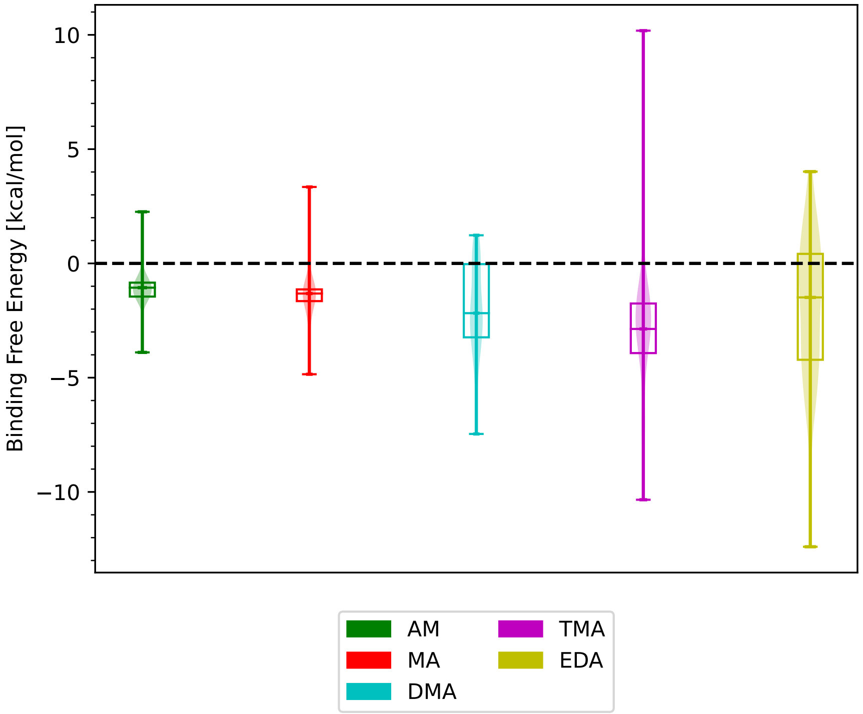

Figure S10: ZORA-DLPNO-CCSD(T_0)/TZVPP//ωB97X-D3BJ/aug-cc-pVTZ-PP binding free energies for the generated structures. The full distribution of binding free energies is plotted together with the distributions for dimers containing each base.

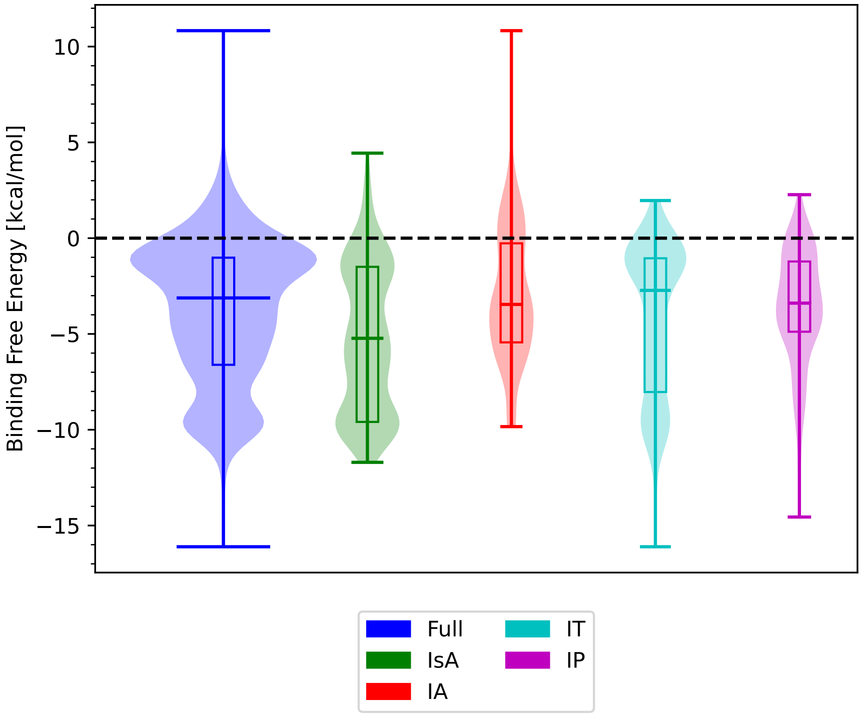

Figure S11: ZORA-DLPNO-CCSD(T_0)/TZVPP//ωB97X-D3BJ/aug-cc-pVTZ-PP binding free energies for the generated structures. The full distribution of binding free energies is plotted together with the distributions for dimers each iodine-containing compound.

Table S9: Lowest binding free energy of iodine containing dimers, given in kcal/mol, calculated at the ZORA-DLPNO-CCSD(T_0)/TZVPP//ωB97X-D3BJ/aug-cc-pVTZ-PP level of theory at 283.15 K and 1 atm. Rows denote the first component, and column denote second component.

	HIO_2	HIO_3	I_2O_4	I_2O_5
HIO_2	−10.7	−10.5	−10.9	−8.8
HIO_3	–	−6.5	−10.3	−7.5
I_2O_4	–	–	−17.0	−15.4
I_2O_5	–	–	–	−12.1
SA	−12.3	−10.3	−11.1	−8.8
MSA	−10.5	−6.8	−8.5	−5.5
NA	−5.8	−1.0	−12.3	−1.7
FA	−7.3	−4.5	−10.4	−4.0
W	−1.1	−1.5	−2.0	−1.0
AM	−2.0	−4.4	−1.8	−1.6
MA	−2.2	−4.3	−3.1	−5.4
DMA	−2.7	−7.8	−5.3	−8.1
TMA	−3.9	−7.9	−2.6	−11.0
EDA	−5.0	−6.9	−6.6	−13.2

References

(1) O. Dohn, A.; B. Moller, K.; P. A. Sauer, S. Optimizing the Structure of Tetracyanoplatinate (II): A Comparison of Relativistic Density Functional Theory Methods. *Curr. Inorg. Chem.* **2013**, *3*, 213–219.
